# The effects of inhaling hydrogen gas on macrophage polarization, fibrosis, and lung function in mice with bleomycin-induced lung injury

**DOI:** 10.1186/s12890-021-01712-2

**Published:** 2021-10-31

**Authors:** Toshiyuki Aokage, Mizuki Seya, Takahiro Hirayama, Tsuyoshi Nojima, Masumi Iketani, Michiko Ishikawa, Yasuhiro Terasaki, Akihiko Taniguchi, Nobuaki Miyahara, Atsunori Nakao, Ikuroh Ohsawa, Hiromichi Naito

**Affiliations:** 1 Department of Emergency, Critical Care and Disaster Medicine, Okayama University Graduate School of Medicine, Dentistry and Pharmaceutical Sciences, 2-5-1 Shikata-cho, Kita-ku, Okayama-shi, Okayama 700-8558 Japan; 2 Department of Disaster Medicine and Management, Okayama University Graduate School of Medicine, Dentistry and Pharmaceutical Sciences, Okayama, Japan; 3 Department of Primary Care and Medical Education, Okayama University Graduate School of Medicine, Dentistry and Pharmaceutical Sciences, Okayama, Japan; 4 Department of Biological Process of Aging, Tokyo Metropolitan Institute of Gerontology, Tokyo, Japan; 5 Department of Emergency, Disaster and Critical Care Medicine, Hyogo College of Medicine, Nishinomiya, Japan; 6 Department of Analytic Human Pathology, Nippon Medical School, Tokyo, Japan; 7 Department of Hematology, Oncology, and Respiratory Medicine, Okayama University Graduate School of Medicine, Dentistry and Pharmaceutical Sciences, Okayama, Japan; 8 Department of Medical Technology, Okayama University Graduate School of Health Sciences, Okayama, Japan

**Keywords:** Acute respiratory distress syndrome, Bleomycin-induced lung injury, Macrophage, Molecular hydrogen, Lung fibrosis

## Abstract

**Background:**

Acute respiratory distress syndrome, which is caused by acute lung injury, is a destructive respiratory disorder caused by a systemic inflammatory response. Persistent inflammation results in irreversible alveolar fibrosis. Because hydrogen gas possesses anti-inflammatory properties, we hypothesized that daily repeated inhalation of hydrogen gas could suppress persistent lung inflammation by inducing functional changes in macrophages, and consequently inhibit lung fibrosis during late-phase lung injury.

**Methods:**

To test this hypothesis, lung injury was induced in mice by intratracheal administration of bleomycin (1.0 mg/kg). Mice were exposed to control gas (air) or hydrogen (3.2% in air) for 6 h every day for 7 or 21 days. Respiratory physiology, tissue pathology, markers of inflammation, and macrophage phenotypes were examined.

**Results:**

Mice with bleomycin-induced lung injury that received daily hydrogen therapy for 21 days (BH group) exhibited higher static compliance (0.056 mL/cmH_2_O, 95% CI 0.047–0.064) than mice with bleomycin-induced lung injury exposed only to air (BA group; 0.042 mL/cmH_2_O, 95% CI 0.031–0.053, *p* = 0.02) and lower static elastance (BH 18.8 cmH_2_O/mL, [95% CI 15.4–22.2] vs. BA 26.7 cmH_2_O/mL [95% CI 19.6–33.8], *p* = 0.02). When the mRNA levels of pro-inflammatory cytokines were examined 7 days after bleomycin administration, interleukin (IL)-6, IL-4 and IL-13 were significantly lower in the BH group than in the BA group. There were significantly fewer M2-biased macrophages in the alveolar interstitium of the BH group than in the BA group (3.1% [95% CI 1.6–4.5%] vs. 1.1% [95% CI 0.3–1.8%], *p* = 0.008).

**Conclusions:**

The results suggest that hydrogen inhalation inhibits the deterioration of respiratory physiological function and alveolar fibrosis in this model of lung injury.

**Supplementary Information:**

The online version contains supplementary material available at 10.1186/s12890-021-01712-2.

## Introduction

Excessive, non-specific inflammation in the lungs initiates pathological processes leading to acute lung injury (ALI) and acute respiratory distress syndrome (ARDS), which directly and indirectly cause destruction of lung tissue including alveolar structures [1]. The development of ARDS is triggered by an immune response that leads to activation of classically activated macrophages, or M1 macrophages, and accumulation of neutrophils in the alveoli. As a result, alveolar epithelial cells and vascular endothelial cells are extensively damaged, and pulmonary edema develops (exudation phase) [2, 3]. Seven to 10 days after onset, proliferation of type II alveolar epithelial cells and fibroblasts is observed in the destroyed alveoli (proliferative phase) [1]. This process of reconstruction is accompanied by persistent inflammation can promote alveolar fibrosis and decrease alveolar compliance (fibrotic phase). Alternatively activated macrophages, or M2 macrophages, which differentiate as a result of persistent inflammation, play a key role in promoting disordered alveolar fibrosis [3]. Overexpression and persistence of M2 macrophages in the alveolar interstitium is a hallmark of the transition to the fibrotic phase [2, 4]. Therefore, novel therapies to reduce persistent inflammation prior to the establishment of irreversible alveolar fibrosis are required and have attracted great interest.

Molecular hydrogen has potent antioxidant and anti-inflammatory properties [5, 6]. The mechanisms underlying the anti-inflammatory effects of hydrogen are becoming clearer with known inhibition of proinflammatory cytokines and upstream signaling molecule [7–9]. Previous studies using animal models suggest that hydrogen may ameliorate hyperoxic lung injury, ovalbumin-induced asthma, and anti-type II collagen antibody-induced arthritis (a model for human rheumatoid arthritis) by inhibiting inflammatory signaling by the innate immune system and regulating signaling cascades that impact macrophages [7, 8, 10]. Inhalation of hydrogen gas may be a straightforward and promising therapeutic option because inhaled gaseous molecules can directly reach the alveoli. Additionally, inhaled hydrogen has a low chemical toxicity [11, 12]. Thus, this gaseous therapy has good clinical feasibility, as long as its flammability can be controlled.

A recent investigation demonstrated that hydrogen inhalation suppressed increases in oxidative stress and inflammation induced by intratracheal bleomycin administration in mice and suppressed the malignant cycle toward lung fibrosis initiated by transforming growth factor (TGF)-β1 and inflammation [13]. In the current study, we sought to expand on those findings and assessed lung physiology using basal respiratory function parameters in the same well-established mouse model of ARDS [14, 15]. Additionally, we examined the upstream mechanisms mediating the protective effects of hydrogen, focusing on macrophage polarization.

## Materials and methods

### Animals

Eight-week-old, C57BL/6 male mice (21–23 g, specific-pathogen free) were purchased from CLEA Japan Inc (Tokyo, Japan). Mice were kept on a 12-h light/dark cycle at 20 to 22 °C and fed sterile food and water. Every effort was made to minimize the number of experimental animals and minimize pain or distress during the experimental procedures. All protocols followed the principles of laboratory animal care (NIH Publication No. 86-23, revised 1985), and all research protocols were reviewed and approved by the Animal Care and Use Committee, Okayama University (OKU-2018876). This study was conducted in compliance with the ARRIVE guidelines (https://arriveguidelines.org/). Animal condition was checked twice daily after the administration of bleomycin. Dying animals that met humane endpoint were euthanized by CO_2_ asphyxiation and were excluded from the analysis. Before scheduled sampling, animals were sacrificed by exsanguination under deep anesthesia with intraperitoneal administration of 0.75 mg/kg medetomidine hydrochloride (Domitor, Meiji Seika Pharma, Tokyo, Japan), 4 mg/kg midazolam (Dormicum, Astellas Pharma, Tokyo, Japan), and 5 mg/kg butorphanol (Vetorphale, Meiji Seika) as previously described [16]. All lobes of the right lung were removed *en bloc*, snap-frozen using liquid nitrogen, and stored at − 80 °C until use. Then, the right lungs were divided, placed in liquid nitrogen, and ground into a powder. Powdered lung tissue (30 mg) was used for RNA extraction for RT-PCR or protein extraction for Western blot. The left lung was used for histopathological analysis.

### Generation of bleomycin-induced lung injury model and inhalation of hydrogen gas

This study was conducted using a well-established mouse model of lung injury and idiopathic pulmonary fibrosis [15, 17]. In summary, lung injury was generated by administrating bleomycin (bleomycin hydrochloride, Nippon Kayaku, Tokyo, Japan) dissolved in saline intratracheally via tracheotomy [17]. The bleomycin causes persistent inflammation pharmacologically in the bronchus and alveoli, eventually resulting in alveolar fibrosis. Mice were anesthetized, and an incision was made through the neck into the front of trachea. Bleomycin dissolved in saline (50 µL, 1 mg/kg) was injected using a Hamilton syringe and a 32G needle, then the wound was closed by cyanoacrylate glue. In sham controls, saline without bleomycin was administrated in the same manner. We conducted a preliminary pathologic assessment, which confirmed that bleomycin administration induced lung injury with temporal changes in pathology that mimicked those observed during ALI/ARDS (Additional file 1: Fig. S1).

Mice were randomly assigned to 1 of 4 experimental groups: (1) saline administration and air inhalation (SA group), (2) saline administration and hydrogen inhalation (SH group), (3) bleomycin administration and air inhalation (BA group), and (4) bleomycin administration and hydrogen inhalation (BH group). A gas cylinder containing 4% hydrogen and 96% nitrogen blended gas was prepared (Taiyo Nissan, Tokyo, Japan). By mixing 800 mL/min of the 4% H_2_/96% N_2_ mixture and 200 mL/min of 100% O_2_ gas, a final mixture of 3.2% H_2_, 20% O_2_ and 76.8% N_2_ gas, delivered at 1 L/min, was generated. Air for the control group is created by mixing 800 mL/min of 100% N_2_ gas and 200 mL/min of 100% O_2_ gas. For gas administration, 5 or fewer mice were placed in a sealed acrylic box (L 40 cm × W 20 cm × H 20 cm) for mixed gas exposure while temperature (acceptable range 22–24 °C) and humidity (acceptable range 40–70%) were monitored. Mice were exposed to either air or 3.2% hydrogen in air for 6 h every day for either 7 or 21 days (Additional file 2: Table S1).

### Respiratory physiological examination

The respiratory physiology was evaluated using a FlexVent^®^ small animal ventilator with spirometer (SCIREQ, Montreal, PQ, Canada). The programs for examination of respiratory function were already programmed into the device and were performed according to the manufacturer’s instructions. Mice were anesthetized as described above, and 1 cm of a 18-gauge endotracheal tube was inserted into the trachea by tracheostomy. The endotracheal tube was attached to the FlexVent. Then, mechanical ventilation is started at 150 respirations per minute, 10 mL/kg of tidal volume, and an inspiratory:expiratory ratio of 2:3 for 1 min. Inspiratory capacity (IC) was measured in mL using the “Deep Inflation” program where the lung was inflated with 27 cm H_2_O of inspiratory pressure. The static compliance (Cst) in mL/cm H_2_O and the static elastance (Est) in cm H_2_O/mL were measured using the “PVs-V” program, where the lungs were inflated stepwise with 40 mL/kg of ventilation volume. Cst and Est were calculated by computer analysis according to the pressure–volume (PV) loop curve created. Total respiratory system resistance (Rs) was measured using the “SnapShot-150” program, where 3 repetitions of sine-wave-pressure forced ventilation (1.2 s, 2.5 Hz) were performed. The protocol consisted of 1 min of mechanical ventilation, 1 cycle of deep inflation, and three 1-min cycles of mechanical ventilation using the Deep Inflation, the PVs-V, and finally the SnapShot-150 programs. IC, Cst and Est were calculated as the median of the three individual measurements.

### Computed tomography

The lung computed tomography (CT) images were taken using a small-animal CT system, Latheta LCT200^®^ (Hitachi, Ltd. Tokyo. JAPAN). The mice are sedated, then inserted into the CT machine and imaged with the following settings: Imaging condition, lung; Pixel size, 48 μm; Slice thickness, 192 μm; Slice interval, 192 μm; X-ray voltage, Low; Scale of tomographic image, − 700 to + 100; and Respiratory synchronization, “Yes”. Of the 70 slices taken of the whole lung field, 40 slices in the center were used for analysis.

After the CT images were saved as JPG files, images of the inside of the thorax were extracted and converted to 8-bit grayscale. ImageJ (National Institutes of Health, Bethesda, Maryland) was used for subsequent image analysis. To trace the areas in the lung containing air, the “Threshold” program was set at “Range: 0–136”. To measure the area, the following settings in “Analyze Particles” program were used: Size (inch^2), 0 -Infinity; Circularity 0.00–1.00; and Show Bare Outline (the obtained area value was defined as A). When this area value A included components which should be excluded, such as pulmonary vessels, a correction was performed. To calculate the area value of the components to be excluded, the "Threshold" program with Range "137–255" and "Analyze Particles" program with Size (inch^2), 0–Infinity; Circularity 0.00–1.00; Show Bare Outline was used, and the obtained area value was defined as B. The area obtained by subtracting B from A was the true air-containing region. The air-containing capacity of the whole lung field was calculated by integrating the slice width and the area containing air as detected above.

### Hematoxylin and eosin and elastica masson staining

The left lung was fixed with 4% paraformaldehyde dissolved in phosphate buffered saline (PBS) for 2 days, embedded in paraffin, then sliced into 4-µm sections. Hematoxylin and eosin (HE) staining and Elastica Masson (EM) staining were performed using standardized protocols by skilled technicians in the Central Research Laboratory at Okayama University. Images were automatically captured using the Nano-Zoomer 2.0RS slide scanner (Hamamatsu Photonics, Shizuoka, Japan) and analyzed using NDP.view2 software, (Hamamatsu Photonics, Shizuoka, Japan). Twenty high-magnification images (total magnification 400×) of HE-stained tissue were captured randomly from one slice, and evaluated using the Lung Injury Score [18] to quantitate the extent of histologic lung injury and the Ashcroft Score [19] to quantitate fibrosis.

### SYBR Green 2-step real-time reverse transcriptase polymerase chain reaction

Messenger RNA levels for interleukin (IL)-6, IL-4, IL-10, IL-13, collagen type I, fibronectin, and ribosomal protein L4 (RPL4) were assessed using SYBR Green, 2-step, real-time, reverse-transcription PCR. RNA extraction was performed with the Nucleospin^®^ RNA kit (Takara Bio Inc., Kusatsu, Japan) using powdered lung tissue (30 mg) according to the manufacturer’s instruction. Total RNA (1 μg) was reverse transcribed with ReverTraAce^®^ qPCR RT Master Mix (TOYOBO Inc., Osaka, Japan). The mixture for SYBR Green PCR was prepared using THUNDERBIRD SYBR qPCR MIX (TOYOBO Inc., Osaka, Japan) and primers (Additional file 2: Table S2). The thermal cycling protocol activated the polymerase for 10 min at 95 °C, followed by 40 cycles of 95 °C for 15 s, and 60 °C for 1 min in a StepOnePlus Realtime PCR machine (Thermo Fisher Scientific, Waltham, Massachusetts).

### Bronchoalveolar lavage fluid assays

Bronchoalveolar lavage fluid (BALF) was collected by injecting 1 mL of PBS into the trachea, flushing it in and out 5 times, and then collecting the fluid. After centrifuging at 500 × *g* at 4 °C, the supernatant was collected, and the total protein concentration was measured via bicinchoninic acid (BCA) assay using the Pierce BCA Protein Assay Kit (Thermo Scientific, Waltham, Massachusetts). Turk's staining solution (200 μL, Nakarai tesque, Kyoto, Japan) was added to the precipitate to stain leukocytes, and the cells were counted using a Burker-Turk counter. To examine IL-6 expression in alveolar macrophages, RT-PCR of BALF cells was performed. Cells were collected by centrifugation from BALF, re-suspended in the lysis buffer included in the Nucleospin XS^®^ RNA kit (Takara Bio Inc., Kusatsu, Japan), and immediately frozen by liquid nitrogen. Then, RNA was extracted according to the manufacturing instructions. The amount of total RNA was 70–100 ng per sample. cDNA was obtained by reverse transcription with ReverTraAce^®^ qPCR RT Master Mix, and real-time PCR for IL-6 was performed.

### Western blotting

Powdered frozen graft tissue (30 mg) was mixed with 300 μL of radioimmunoprecipitation assay (RIPA) buffer, which is consisted of 50 mM Tris–HCl (pH 8.0), 150 mM NaCl,1% Igepal^®^ CA-630 (Merck, Darmstadt, Germany), 0.5% Sodium deoxycshoate, 0.1% sodium dodecyl sulfate (SDS) and 1 mM EDTA, and cOmplete™ Mini Protease Inhibitor Cocktail (Merck. Darmstadt, Germany). After homogenizing and measuring the protein concentration, samples were further dissolved in SDS-PAGE sample buffer (62.5 mM Tris–HCl pH 6.8, 10% glycerol, 2% SDS, bromophenol blue) to 1 μg/μL.

For the analysis of collagen type I (COL1), fibronectin and α-smooth muscle actin (αSMA), proteins (10 μg) from lung tissue were separated by electrophoresis on 8% acrylamide gels without SDS and transferred to Immobilon^®^-P polyvinylidene difluoride (PVDF) membrane (0.45 µm) (Merck, Darmstadt, Germany). For the analysis of TGFβ, proteins (10 μg) from lung tissue were separated by electrophoresis on 12% acrylamide, 0.1% SDS gels.

PVDF membranes are blocked with 5% non-fat dry milk to prevent non-specific binding of antibodies. Primary antibody against fibronectin, αSMA, COL1, and TGFβ were diluted with Can Get Signal immunoreaction enhancer solution 1 (Toyobo, Osaka, Japan) (Additional file 2: Table S3), and incubated with the membranes overnight at 4 °C. Horseradish-peroxidase–conjugated secondary antibodies against mouse IgG and rabbit IgG were diluted with Can Get Signal immunoreaction enhancer solution 2 (Toyobo, Osaka, Japan) and membranes were incubated for 2 h at room temperature. Chemiluminescence detection was performed with ECL Prime Western Blotting Detection Reagents (Cytiva, Tokyo, Japan) and a WSE-6100 LuminoGraph I (ATTO Corporation, Tokyo, Japan).

### Immunohistochemistry

Paraffin-embedded lung tissue sections (4 µm) were immunostained for TGF-β and IL-6 using an ABC Kit (Vector laboratories INC., Burlingame, California). Information on the primary and secondary antibodies used is provided in Additional file 2: Table S3. Sections were deparaffinized, rehydrated, and treated for antigen retrieval with 10 mM citric acid pH 6.0 at 120 °C for 10 min in a pressure cooker. Endogenous peroxidase inhibition was performed with 0.3% hydrogen peroxide in PBS for 20 min at room temperature. Blocking treatment was performed with 10% goat serum in tris buffered saline with 0.1% Tween 20 (TBS-T) to prevent non-specific binding of antibodies. The primary antibodies were diluted by Can Get Signal immunostaining Solution A (Toyobo, Osaka, Japan), applied to the sections, incubated overnight at 4 °C, and then washed with TBS-T. Biotin-conjugated secondary antibodies were diluted by Can Get Signal immunostaining Solution A, applied on the sections, and incubated for 2 h at room temperature. After washing, ABC reagent was applied to the sections then incubated for 30 min at room temperature as per the manufacturer’s instructions. For 3,3′-diaminobenzidine (DAB) staining, one DAB tablet (10 mg per tablet, FUJIFILM Wako Pure Chemical Corporation, Osaka, Japan) was dissolved in 50 mL of 0.05 mol/L Tris–HCl buffer pH 7.6 with 10 μL of 30% hydrogen peroxide as per the manufacturer’s instructions. Sections were incubated in DAB solution for 10 min at room temperature, then washed under running water, counterstained with hematoxylin, dehydration, clearing, and coverslipping.

### Immunofluorescence

Paraffin blocks were sectioned, deparaffinized, rehydrated, and treated for antigen retrieval using the technique described above. The multiplex fluorescent immunostaining was used for staining with anti-ionized calcium binding adaptor molecule 1 (Iba-1) antibody and anti-CD163 antibody. Information on primary and secondary antibodies is provided (Additional file 2: Table S3). Blocking treatment was performed with Super Block^®^ (SCY AAA125, Cosmo Bio Co., Ltd. Tokyo, Japan). Anti-Iba-1 antibody and anti-CD163 antibody were diluted in Can Get Signal immunostaining Solution A, then incubated on the tissue section overnight at 4 °C. After washing, the sections were incubated with fluorescently labeled secondary antibodies with Alexa Flour (AF) 488 or 594. DAPI-Fluoromount G^®^ (0100-20, SouthernBiotech, Birmingham, AL) was used for nuclear staining and sealing.

Fluorescent images were taken by the Mantra™ Quantitative Pathology Imaging System (PerkinElmer Inc., Waltham, Massachusetts), and cells were counted in the alveoli and interstitium were automatically using the InForm^®^ 2.4.10 software (Akoya Biosciences, Inc., Menlo Park, California). Three images were taken randomly from each section with a 200× image. Fluorescence imaging was performed at 488 nm, and 594 nm wavelengths. In the InForm software, a computer learning system was used to learn the characteristics of alveolar epithelium and alveolar interstitum tissues and exclude tracheal epithelial cells. The cells were identified by DAPI staining, and the immunostaining was visualized at 488 nm (Iba-1) or 594 nm (CD163) wavelength. The intensity thresholds for Iba-1-positive and CD163-positive cells were carefully adjusted and identified, and all images were analyzed according to the same rules.

### Statistics

Statistical analysis was performed using IBM SPSS Statistics version 23.0 (IBM, Armonk, New York). Statistically significant differences between groups were determined using an unpaired two-tailed Student’s t-test for single comparisons; the Kruskal–Wallis test followed by Dunn's multiple comparison test for multiple comparisons. All values are presented as mean ± 95% confidence interval (CI). Results were considered significant at *p* < 0.05.

## Results

### Hydrogen inhalation for 21 days mitigates respiratory physiological dysfunction during fibrotic phase after bleomycin-induced lung injury

**Fig. 1:**
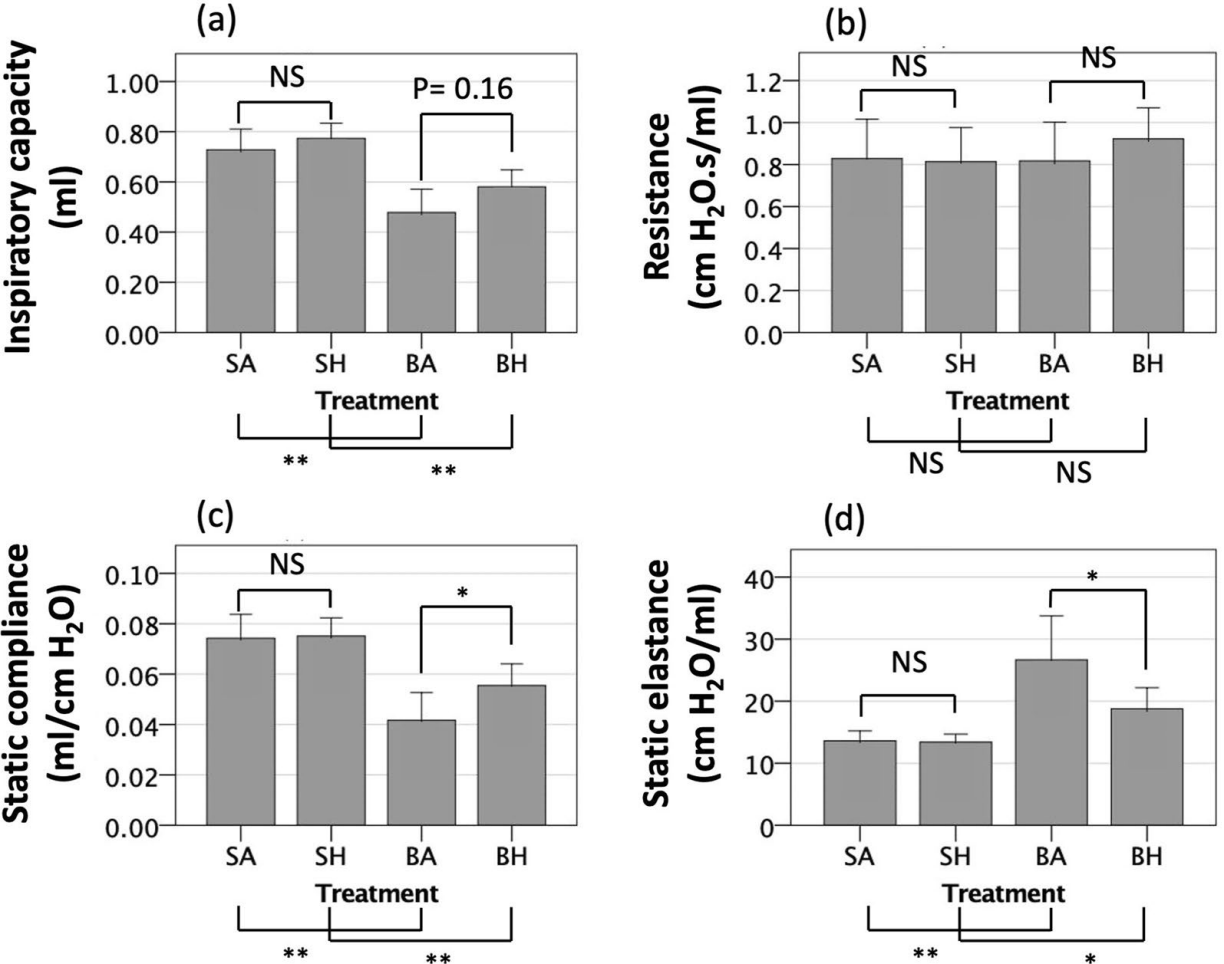
The results of respiratory physiological examination of respiratory function during the fibrotic phase of bleomycin-induced lung injury. **a** Inspiratory capacity (IC). **b** Total respiratory system resistance (Rs). There were no differences in Rs among the four groups (*p* = 0.51). **c** Static compliance (Cst). **d** Static elastance (Est). The BA and BH groups had lower IC and Cst and higher Est than the SA and SH groups; the BH group had significantly higher Cst and lower Est than the BA group. SA, saline administration and air inhalation, n = 6; SH, saline administration and hydrogen inhalation, n = 6; BA, bleomycin administration and air inhalation, n = 11; BH, bleomycin administration and hydrogen inhalation, n = 11. NS, not significant; *, *p* < 0.05; **, *p* < 0.01; error bars indicate 95% CI

While IC and Rs were not significantly different between mice that received hydrogen therapy and mice that received sham/air therapy (BH group vs. BA group, Fig. 1a, b) when we examine respiratory physiology, Cst, an index of the distensibility of the respiratory system, was significantly higher in mice that received hydrogen therapy (BH 0.056 mL/cm H_2_O [95% CI 0.47–0.64] vs. BA 0.042 mL/cm H_2_O [95% CI 0.031–0.053], *p* = 0.02) (Fig. 1c). The Est of the lungs in mice that received hydrogen therapy after lung injury was significantly lower than in mice with air therapy (BH 18.8 cm H_2_O/mL [95% CI 15.4–22.2] vs. BA 26.7 cm H_2_O/mL [95% CI 19.6–33.8], *p* = 0.02) (Fig. 1d). In fibrotic phase after lung injury, fibrotic changes progress in the alveolar interstitum, and the lung tissues become hardened. These results suggests that hydrogen inhalation therapy preserved the ability of the lung to expand and reduced lung stiffness. There were no differences in any of the respiratory parameters examined between hydrogen and sham/air therapy when lung injury was not induced (SH and SA groups), suggesting that hydrogen has no effects on respiratory physiological function in individuals without alveolar damage (Fig. 1).

### Hydrogen inhalation for 21 days can attenuate the reduction in lung capacity typical of bleomycin-induced lung injury

**Fig. 2:**
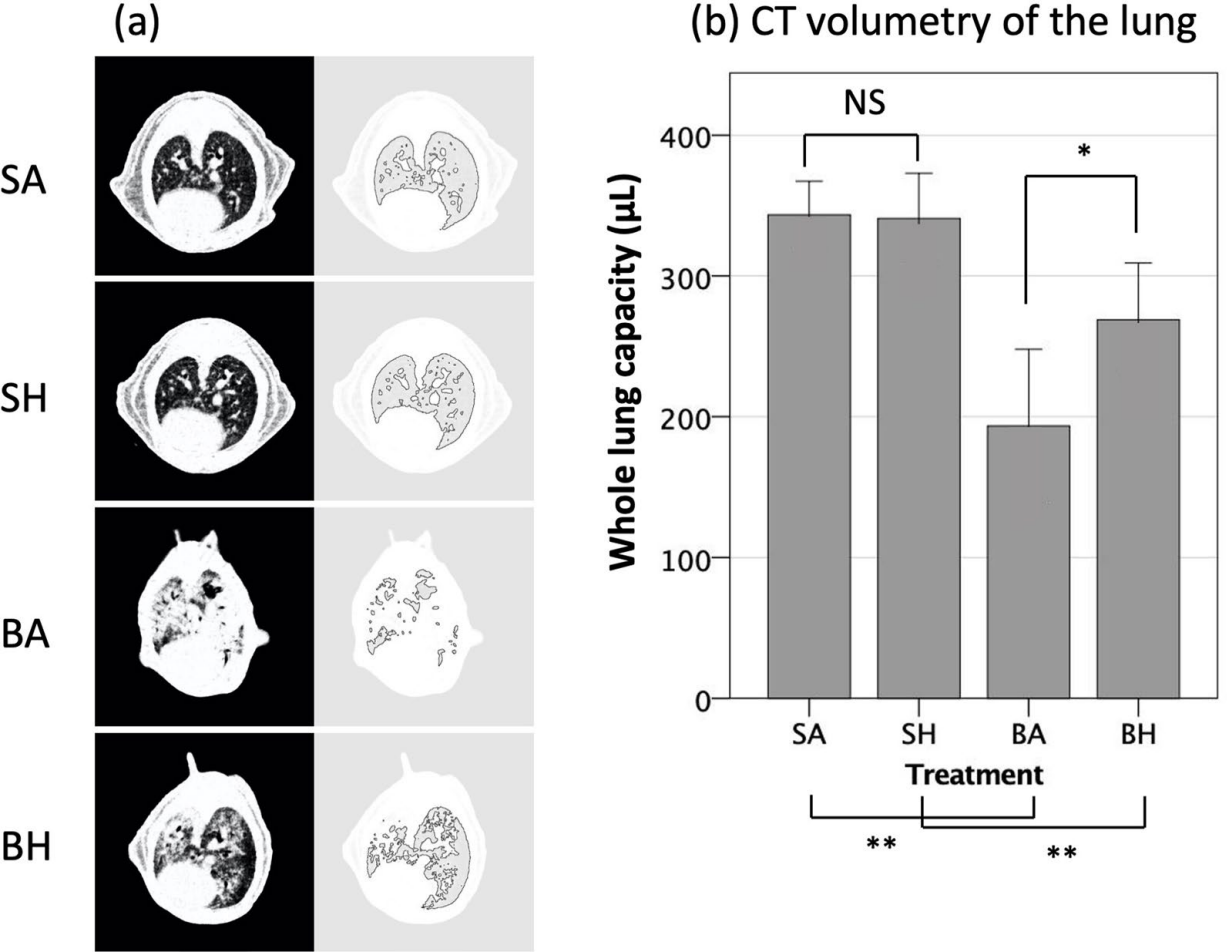
Volumetry evaluated using computed tomography. **a** Left column. CT images of the thoracic cavity at the level of the left ventricle. Right column. Representative images obtained using "Analyze Particles" program. The "Bare Outline" is indicated by the black line. The area of the aerated field, that enclosed within the black lines, was analyzed with ImageJ. **b** The aerated volume was calculated by integrating the aerated area. The aerated volume was reduced by bleomycin-induced lung injury, and inhaling hydrogen preserved the aerated volume after bleomycin-induced injury. SA, saline administration and air inhalation, n = 6; SH, saline administration and hydrogen inhalation, n = 6; BA, bleomycin administration and air inhalation, n = 9, BH, bleomycin administration and hydrogen inhalation, n = 9. NS, not significant; *p < 0.05, **p < 0.01. Error bars: 95% CI

Although bleomycin-induced lung injury significantly reduced aerated lung capacity as determined using CT volumetry (BA group), hydrogen treatment for 21 days significantly ameliorated this reduction as indicated by higher aerated lung capacities in BH group than those in the BA group (BH 269 μL [95% CI 228–309] vs. BA 193 μL [95% CI 139–248], *p* = 0.02). Hydrogen treatment had no effect on aerated lung capacity in the absence of lung injury (SH and SA groups) (Fig. 2a, b).

### Hydrogen inhalation for 21 days reduced alveolar fibrosis after bleomycin-induced lung injury

**Fig. 3:**
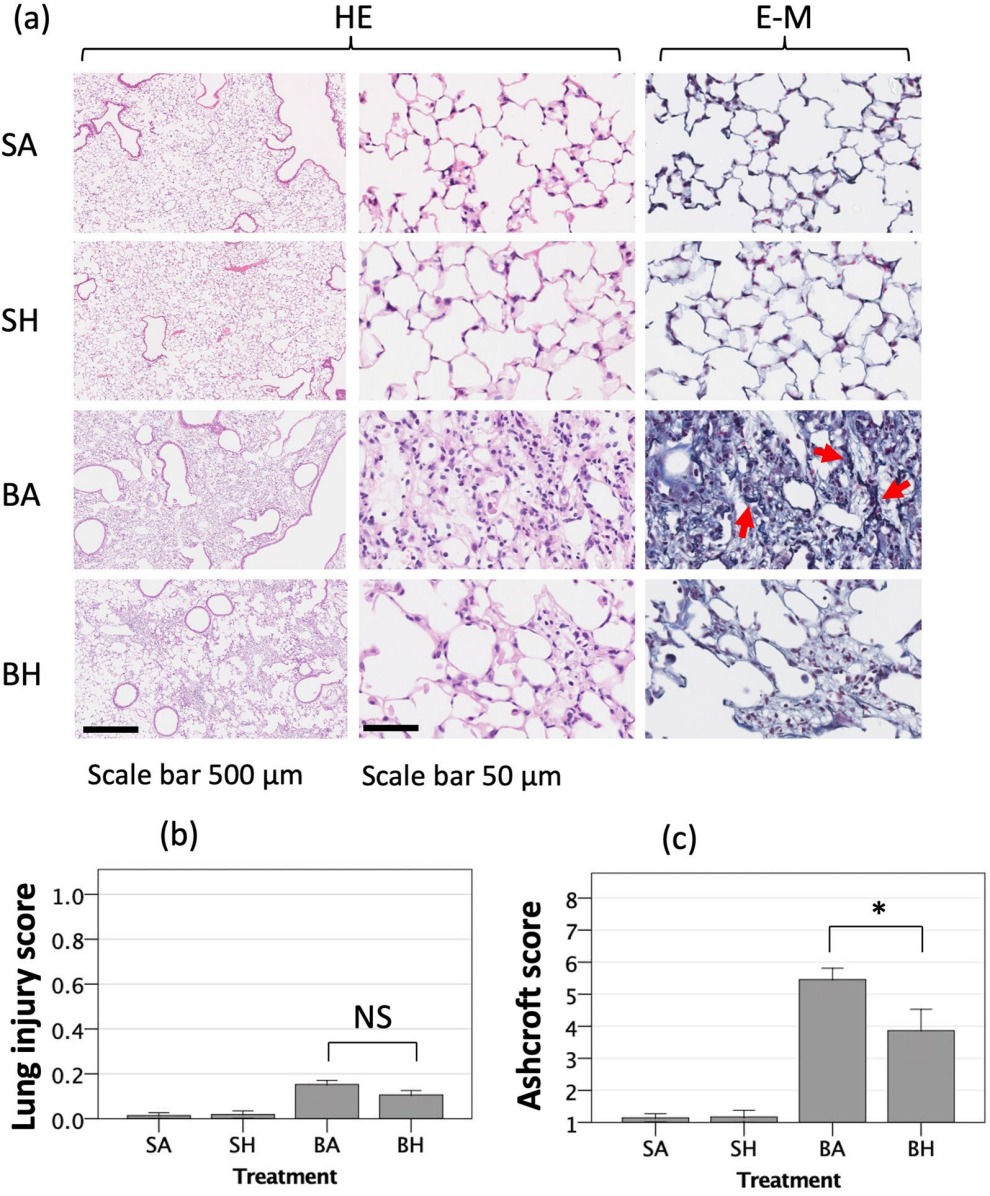
Examination of alveolar fibrosis in lung tissue. **a** Representative images of bleomycin-induced alternations in the alveolar interstitum and bronchial epithelium are shown using hematoxylin and eosin (HE) staining (×12.5 and ×200 images) and Elastica Masson (E-M) staining (×200 image). Extracellular matrix component proteins appear as blue–purple in E-M staining. Red arrows indicated particularly thick collagen bundles, which stain as black-purple bundles. Inhalation of hydrogen gas suppressed fibrous tissue production after lung injury. **b** Lung injury scores were low overall and not significantly different between treatment groups. **c** Ashcroft scores clearly demonstrated that there were more fibrotic changes in the lungs of the BA group as compared with the BH group. SA, saline administration and air inhalation, n = 6; SH, saline administration and hydrogen inhalation, n = 6; BA, bleomycin administration and air inhalation, n = 11, BH, bleomycin administration and hydrogen inhalation, n = 11. *p < 0.05; error bars indicate 95% CI

There were many cytoplasm-rich cells, which might include fibroblasts, myofibroblasts, and inflammatory cells, in the alveolar interstitium in lungs with bleomycin-induced lung injury. The presence of these cells in the alveolar interstitium was attenuated by hydrogen treatment (BH group) (Fig. 3a, H&E staining). Collagen bundles were seen in the interstitium of the mice with bleomycin-induced lung injury (BA group) and were less frequently observed in mice treated with hydrogen for 21 days (BH group) (Fig. 3a, E-M). Lung injury scores of the lungs of mice in the BA and BH groups were as low as 0.12, and no statistically significances differences were seen between the treatment groups (Fig. 3b). Ashcroft score, which quantitated the extent of fibrosis, demonstrated that there were more fibrotic changes in the lungs of mice in the BA group compared with the BH group (Fig. 3c).

### Hydrogen inhalation for 21 days inhibited increases in fibronectin protein expression after bleomycin-induced lung injury

**Fig. 4:**
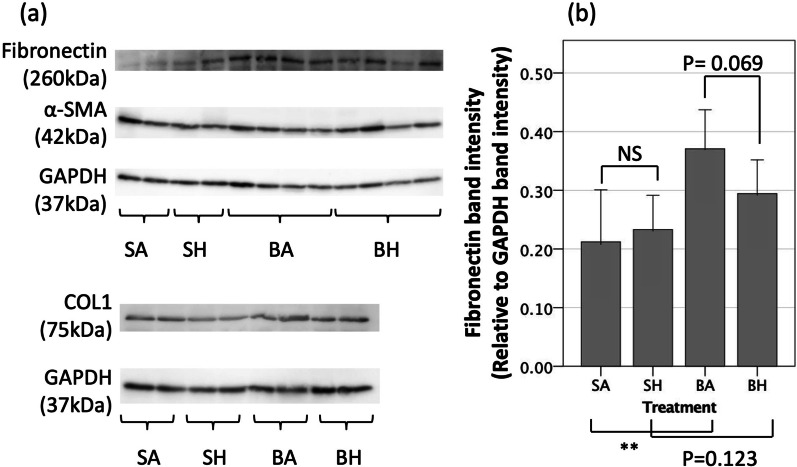
Western blot evaluation of extracellular matrix component proteins. **a** Western blot evaluation of fibronectin, type 1 collagen (COL1), alpha-smooth muscle actin (αSMA) and glyceraldehyde 3-phosphate dehydrogenase (GAPDH). The original, full-length blot images are shown in Supplementary Information. **b** The band intensity of fibronectin evaluated as a ratio to GAPDH. Inhaling hydrogen gas may decrease the production of fibronectin in lung (*p* = 0.069). SA, saline administration and air inhalation, n = 6; SH, saline administration and hydrogen inhalation, n = 6; BA, bleomycin administration and air inhalation, n = 11, BH, bleomycin administration and hydrogen inhalation, n = 11. NS: not significant, **p < 0.01. Error bars: 95% CI

Expression of COL1 and αSMA did not differ between any of the treatment groups at the 21-day timepoint. (Fig. 4a, Additional file 1: Fig. S2, Additional file 3). Fibronectin was more highly expressed after bleomycin-induced lung injury in the BA group than in the SA group (BA 0.371 [95% CI 0.314–0.428] vs. SA 0.212 [95% CI 0.143–0.281], *p* = 0.004), and hydrogen therapy showed a tendency to reduce this upregulation, though the differences in protein levels did not reach statistical significance (BH 0.294 [95% CI 0.245–0.343] vs. BA 0.371 [95% CI 0.314–0.428], *p* = 0.069) (Fig. 4b).

### Hydrogen inhalation for 7 days attenuates the upregulation of critical interleukins and downregulates fibronectin mRNA in lung tissue after bleomycin-induced lung injury

**Fig. 5:**
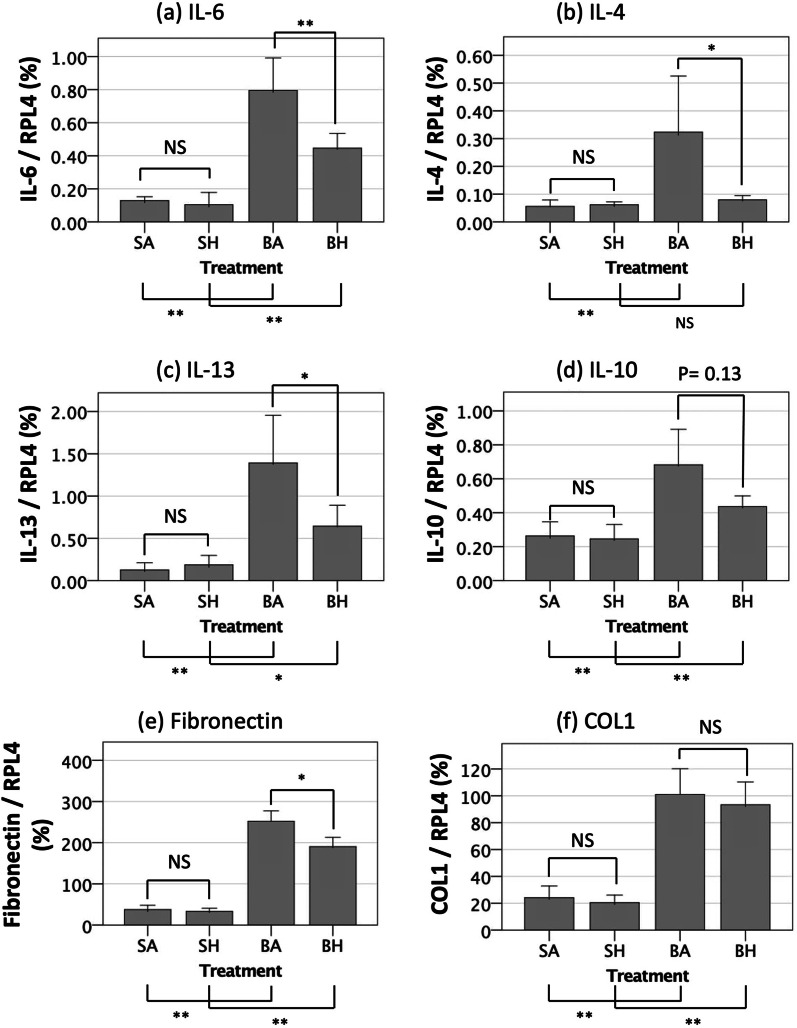
mRNA expression levels of **a** interleukin (IL)-6, **b** IL-4, **c** IL-13, **d** IL-10, **e** fibronectin and **f** type 1 collagen (COL1) were measured using SYBR Green 2-step real-time reverse transcriptase polymerase chain reaction (RT-PCR). Inhaling hydrogen gas significantly suppressed the expression of IL-6, IL-4, and IL-13 induced by bleomycin administration. The expression of fibronectin is significantly reduced by hydrogen inhalation. SA, saline administration and air inhalation, n = 12; SH, saline administration and hydrogen inhalation, n = 12; BA, bleomycin administration and air inhalation, n = 28, BH, bleomycin administration and hydrogen inhalation, n = 28. NS: not significant, *p < 0.05, **p < 0.01. Error bars: 95% CI

To begin to discern the mechanisms underlying hydrogen’s protective effects in reducing fibrosis in this animal model, we examined the inflammatory response 7 days after lung-injury induction. Expression of the mRNAs for IL-6, IL-4 and IL-13, all of which are considered pro-inflammatory cytokines in the lung, were significantly upregulated 7 days after bleomycin administration as compared with saline-treated control lungs. Repeated hydrogen inhalation significantly suppressed upregulation of IL-6, IL-4 and IL-13 in response to bleomycin-induced lung injury (Fig. 5a, b, c). There was no statistical difference in IL-10 mRNA expression between lungs from mice in the BA and BH groups (*p* = 0.13) (Fig. 5d).

The mRNA expression of fibrinogen and COL1, components of the extracellular matrix, were also investigated 7 days after bleomycin treatment. The levels of fibrinogen mRNA were reduced by hydrogen therapy, while the levels of COL1 mRNA were not (Fig. 5e, f).

### Hydrogen inhalation for 7 days suppresses the expression of TGF-β in the alveolar interstitium after bleomycin-induced lung injury

**Fig. 6:**
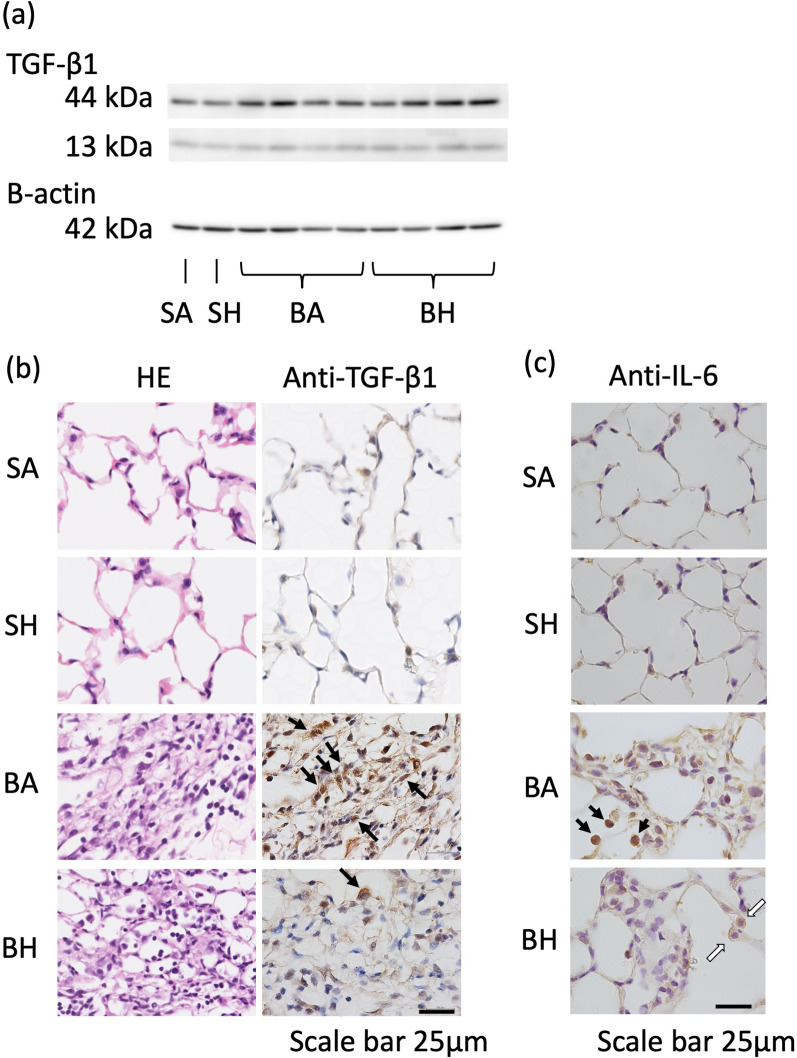
TGF-β1 expression. **a** TGF-β1 protein in the lung as examined by western blotting. Beta-actin was evaluated as a housekeeping protein. There are two TGF-β1 bands, 44 kDa and 13 kDa. The 13 kDa band represents active form. There was no difference of 13 kDa band intensity among the 4 groups. The original, full-length blot images are shown in Supplementary Information. **b** Immunostaining for TGF-β1 to localize protein expression in the alveoli. Black arrows indicate cells strongly positive for TGF-β1. SA, saline administration and air inhalation; SH, saline administration and hydrogen inhalation; BA, bleomycin administration and air inhalation; BH, bleomycin administration and hydrogen inhalation. **c** Immunostaining with IL-6 antibody was performed on lung sections obtained from mice on day 7 after bleomycin administration. Black and white arrows indicate alveolar macrophages in BA and BH groups respectively

When TGF-β1 protein expression was analyzed by Western blotting, hydrogen treatment did not affect TGF-β1 expression (Fig. 6a, Additional file 3). However, when the localization of TGF-β1 was examined using immunostaining, fewer TGF-β1-positive cells were found in the alveolar interstitium after hydrogen therapy than in sham/air treated controls (Fig. 6b). Immunostaining for IL-6 demonstrated that majority of IL-6-producing cells in the lung were alveolar macrophages (Fig. 6c).

### Hydrogen inhalation for 7 days reduced the expression of IL-6 in cells in the BALF

**Fig. 7:**
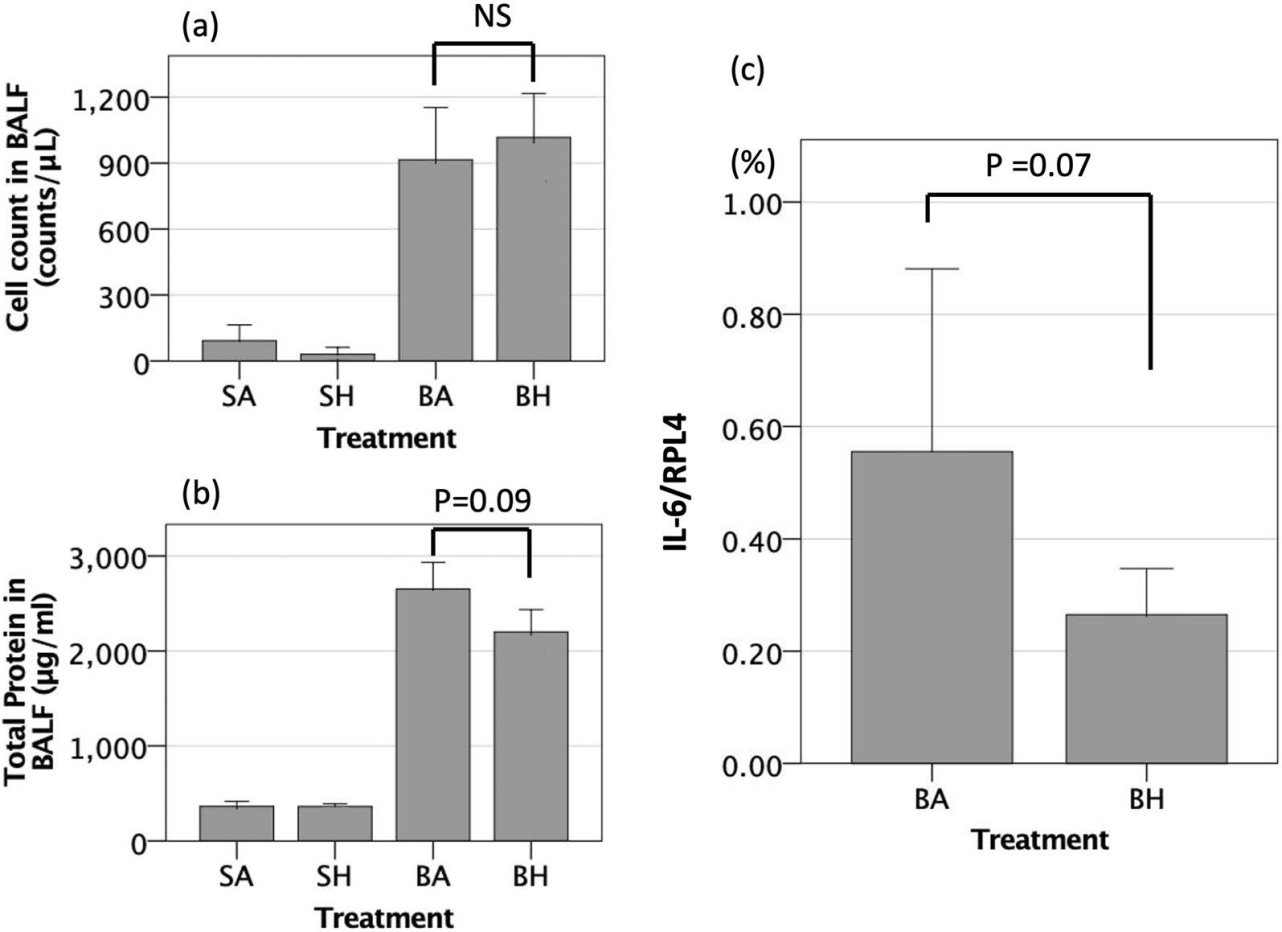
Bronchoalveolar lavage fluid (BALF) assay. **a** Inhaled hydrogen therapy did not affect the number of cells in the BALF. **b** Hydrogen therapy tended to decrease the protein concentration in the BALF (*p* = 0.09). SA, saline administration and air inhalation, n = 6; SH, saline administration and hydrogen inhalation, n = 6; BA, bleomycin administration and air inhalation, n = 15, BH, bleomycin administration and hydrogen inhalation, n = 15. NS, not significant; Error bars: 95% CI. **c** mRNA expression levels of IL-6 in isolated cells from BALF. The median values of IL-6/RPL4 mRNA tended to be higher in the BA group as compared with the BH group (*p* = 0.07). BA, bleomycin administration and air inhalation, n = 10, BH, bleomycin administration and hydrogen inhalation, n = 10. Error bars: 95% CI

Hydrogen inhalation did not alter the number of cells in the BALF (BA 969 cells/μL [95% CI 776–1163] vs. BH 1017 cells/μL [95% CI 843–1190], *p* = 0.74) (Fig. 7a). Hydrogen inhalation also did not reduce the protein concentration in the BALF after bleomycin insult (BA 2567 μg/mL [95% CI 2304–2830] vs. BH 2258 μg/mL [95% CI 2047–2469], *p* = 0.09) (Fig. 7b).

Real-time RT-PCR using the cells isolated from BALF revealed upregulation of IL-6 mRNA 7 days after bleomycin insult. Hydrogen inhalation attenuated this IL-6 upregulation (Fig. 7c).

### Hydrogen inhalation for 7 days reduces M2-biased macrophages in the alveolar interstitium after bleomycin-induced lung injury

**Fig. 8:**
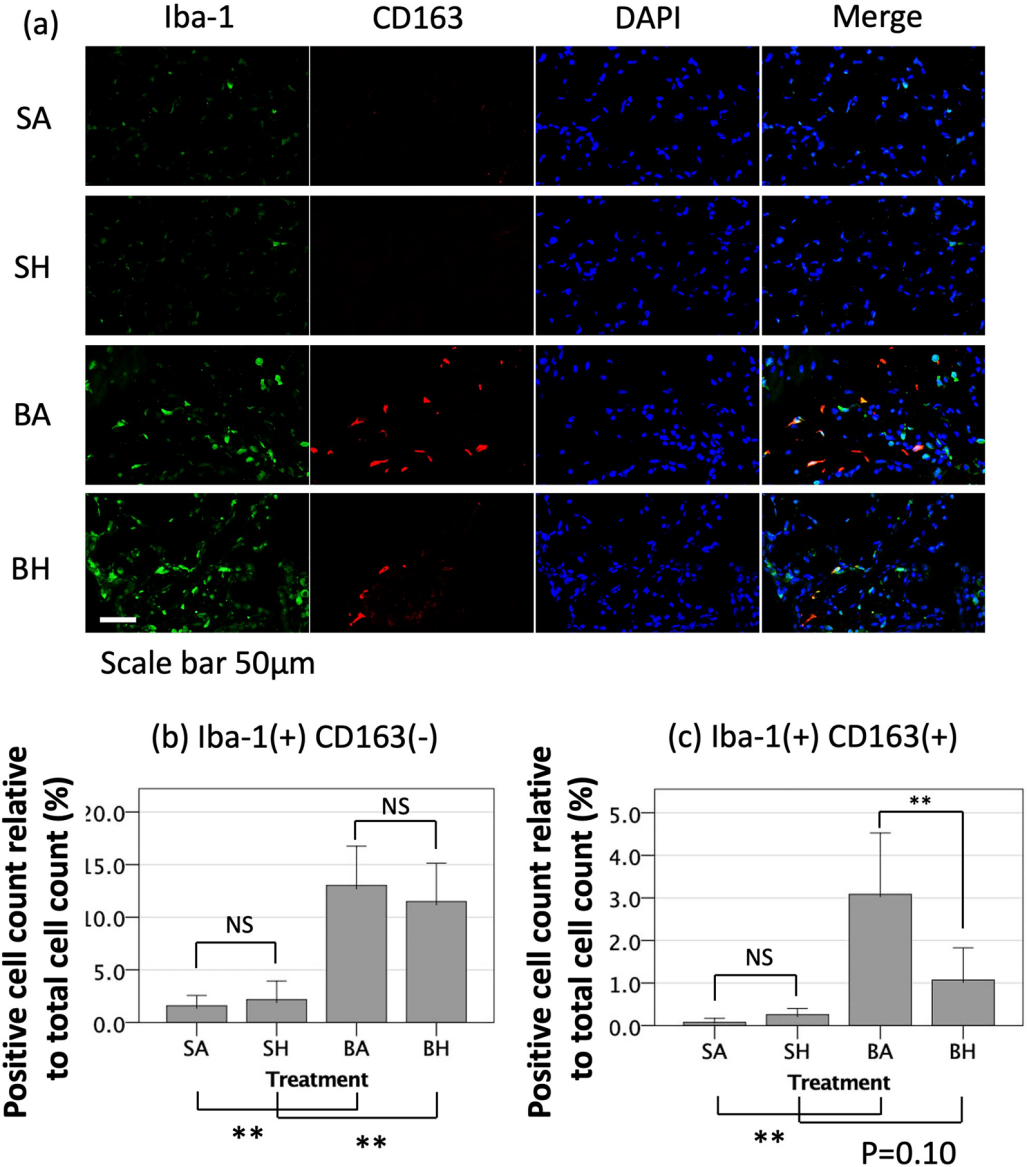
Immunofluorescent localization of macrophage markers. **a** Anti-Iba-1 was used to detect total macrophages, and anti-CD163 was used to detect M2-biased macrophages. Representative images showing DAPI (blue), Iba-1 (Green, AlexaFlour488), and CD 163 (red, AlexaFlour594). **b** Quantitation of Iba-1-positive, CD163-negative cells (all macrophages except M2 macrophages). **c** Quantitation of Iba-1-positive, CD163-positive cells (M2-biased macrophages). The graphs show the percentage obtained by dividing the number of macrophages of the target phenotype by the total number of cells (measured by DAPI staining). There was no difference in the number of Iba-1-positive and CD163-negative cells between the groups with and without hydrogen inhalation; however, the number of Iba-1-positive and CD163-positive cells was significantly reduced by inhaling hydrogen for 7 days. SA, saline administration and air inhalation, n = 6; SH, saline administration and hydrogen inhalation, n = 6; BA, bleomycin administration and air inhalation, n = 15, BH, bleomycin administration and hydrogen inhalation, n = 15. NS: not significant, *p < 0.05, **p < 0.01. Error bars: 95% CI

To identify the phenotype of macrophages in the alveoli and alveolar interstitum, anti-Iba-1 antibody was used to detect all macrophages, and anti-CD163 antibody was used to specifically detect M2-biased macrophages. There was an increase in the number of Iba-1-positive, CD163-negative macrophages in the alveoli of the lung 7 days after bleomycin insult, but hydrogen treatment did not alter the number of Iba-1-positive, CD163-negative cells present (BA 13.0% [95% CI 9.3–16.8%] vs. BH 11.5% [95% CI 7.8–15.1%], *p* = 0.74) (Fig. 8a, b). In contrast, bleomycin-induced lung injury increased of the number of Iba-1-positive, CD163-positive macrophages in the alveoli, indicating that more M2-biased macrophages were present in the tissue, and hydrogen therapy significantly decreased the presence of these M2-biased macrophages in the alveoli (BA 3.1% [95% CI 1.6–4.5%] vs. BH 1.1% [95% CI 0.3–1.8%], *p* = 0.008) (Fig. 8a, c).

## Discussion

Hydrogen inhalation therapy has been proven effective in mitigating in several animal models of lung injury including hyperoxic lung injury, hemorrhagic shock-induced lung injury, radiation-induced lung injury, and bronchial asthma [7, 8, 20, 21]. Our study is the first to prove that hydrogen inhalation therapy effectively attenuates the decline of respiratory physiological function induced by bleomycin in a mouse model of persistent lung inflammation and fibrosis. To the best of our knowledge, this is the first study to assess the effects of hydrogen inhalation using physiologic respiratory function tests and CT volumetry. We also demonstrated that the protective effects of hydrogen gas inhalation therapy in this lung injury model were accompanied by attenuation of pro-inflammatory cytokine expression and reduced the number of M2-biased macrophages in the lung after bleomycin-induced lung injury).

In this study, we administered an air mixture with 3.2% hydrogen for 6 h daily beginning on the day of bleomycin administration and continuing for 21 days. Other investigations of inhaled hydrogen therapy have shown effective results with hydrogen concentrations of 2–4% [5, 7, 20–24], and repeated inhalation was reported to be more effective than continuous inhalation in rat model of Parkinson’s disease [25]. Because the endpoint of this study was to assess lung function during the fibrotic phase after lung injury, we targeted treatment during the proliferative phase of ARDS, which ordinary occurs 7 to 21 days after the onset of lung injury [1]. Our preliminary studies indicated that the bleomycin-induced lung injury model mimicked the temporal changes in pathology observed during ARDS and dictated our protocol of repeated hydrogen inhalation for 21 days after bleomycin administration.

During ARDS, pathogen-associated molecular pattern molecules and damage-associated molecular pattern molecules stimulate type II alveolar epithelial cells and alveolar macrophages to secrete pro-inflammatory cytokines. The permeability of pulmonary capillaries and alveolar epithelial cells increases, and exudate flows from the blood vessels into the alveoli and interstitium. The immune cells responsible for this are classically activated macrophages (M1 macrophages) and neutrophils [1]. IL-6, which is expressed in type II alveolar epithelial cells and M1 macrophages, is associated with lung fibrosis through the polarization of M2 macrophages [26–30]. Although M2 macrophages are important for tissue repair, an excess of M2 macrophages can cause organ fibrosis [31, 32]. Suppression or deletion of IL-6 suppresses M2 macrophage polarization and attenuates lung fibrosis [33, 34]. In our study, immunostaining showed the expression of IL-6 was induced in alveolar macrophages by bleomycin administration. Although it is difficult to differentiate between the IL-6 produced by the cells and that bound to cells, IL-6 was most likely secreted by alveolar cells considering upregulation of IL-6 in BALF cells. Our finding that hydrogen inhalation therapy reduced IL-6 mRNA expression is consistent with published work demonstrating that hydrogen suppresses the expression of IL-6 [7, 20, 35–41]. Therefore, it is reasonable to speculate that the reduction in IL-6 expression in response to hydrogen inhalation therapy caused less M2-biased macrophage polarization and less fibrosis after bleomycin-induced lung injury.

IL-4 and IL-13 also induce the polarization of macrophages to M2 macrophages, which decreases inflammation and encourages tissue repair. Persistent or excessive expression of IL-4 or IL-13 and the accompanying M2 macrophage polarization leads to abnormal organ fibrosis [31, 32]. In our study, the expression of IL-4 and IL-13 mRNAs were decreased by hydrogen inhalation therapy. The regulation of IL-4 and IL-13 by hydrogen may result in an anti-fibrotic effect through suppression of M2-biased macrophage polarization and thereby reduce alveolar fibrosis. TGF-β is secreted from M2 macrophages [42]. Hydrogen administration decreased the number of TGF-β-positive cells in the alveolar interstitium, again indicating that control of M2-biased macrophage polarization may be an important mechanism underlying the therapeutic benefits of inhaled hydrogen. However, as another interpretation, the decrease in TGF-β1 positive cells by immunostaining could also be the result of downregulation of TGF-β1 production in mesenchymal cells and/or epithelial cells by hydrogen gas inhalation, reflected in the decrease in TGF-β1 binding to TGF-β1 receptors on macrophages in the interstitum. The results of this study support models put forth by others that hydrogen therapy regulates upstream signals in a cascade impacting macrophages and innate immunity that leads to inflammation [7, 8, 10, 43].

The study has some limitations. The pathogenesis of lung injury in clinical practice is diverse. Bleomycin-induced lung injury is only one type of drug-induced lung injury and does not replicate all possible lung injury presentations. Second, determining the most effective hydrogen administration regimen—timing, duration and concentration—will be required for clinical application. Third, to identify M2-biased macrophages by immunostaining, we used anti-CD163 antibody, although the expression of CD163 antigen does not necessarily associate with the entire cellular phenotype of M2 macrophage [44]. Finally, the mechanisms by which hydrogen inhibits bleomycin-induced lung injury were not fully elucidated in this study. Hydrogen reduced the number of M2-biased macrophages in the alveolar interstitium with associated inhibition of increases in IL-6, IL-4 and IL-13 that occur after lung injury. Although we observed a convincing impact on M2-biased macrophages in response to hydrogen, currently, we do not have clear evidence that hydrogen directly affects macrophage polarization or fibrotic potential. Moreover, the classification of polarized macrophages is rather complex, and M2 macrophages are divided into subclasses. A study to determine the effects of hydrogen on macrophage polarization, fibrotic potential, or M2 macrophage subclass as a potential underlying mechanism of hydrogen therapy is warranted and may advance our understanding of hydrogen biology.

## Conclusion

Repeated hydrogen inhalation therapy with 3.2% hydrogen for 6 h per day for 21 days inhibited the decline of respiratory physiological function and increase in alveolar fibrosis induced by bleomycin. Hydrogen administration increased ventilation and increased alveolar compliance, which strongly suggests that hydrogen inhalation would improve the clinical profile of ARDS patients when used as therapy.

## Supplementary Information

**Additional file 1: Fig. S1.** Histological changes over time in bleomycin model; **Fig. S2.** The band densitometries of western blotting in COL1 and αSMA.

**Additional file 2: Table S1.** Information on the number and treatments of samples in the analysis; **Table S2.** Primer summery; **Table S3.** Antibody summery.

**Additional file 3.** The original, full-length western blot images of fibronectin, αSMA, GAPDH, COL1, TGF-β1 and β-actin.

## Data Availability

The datasets used and/or analyzed during the current study are available from the corresponding author on reasonable request.

## Abbreviations

ALI: Acute lung injury
ARDS: Acute respiratory distress syndrome
BA: Bleomycin administration and air inhalation
BH: Bleomycin administration and hydrogen inhalation
SA: Saline administration and air inhalation
SH: Saline administration and hydrogen inhalation
IC: Inspiratory capacity
Cst: Static compliance
Est: Static elastance
Rs: Respiratory system resistance
E-M: Elastica Masson
RPL4: Ribosomal protein L4
COL1: Collagen type I
αSMA: α-Smooth muscle actin
Iba-1: Ionized calcium binding adaptor molecule 1
IL: Interleukin
TGF: Transforming growth factor
