# Supplementary material for: The effects of inhaling hydrogen gas on macrophage polarization, fibrosis, and lung function in mice with bleomycin-induced lung injury
Source: BMC Pulm Med. 2021 Oct 31;21:339. doi: 10.1186/s12890-021-01712-2 (PMC8559370; doi:10.1186/s12890-021-01712-2)
Supplement: Supplementary file 1 — Additional file 1: Fig. S1. Histological changes over time in bleomycin model; Fig. S2. The band densitometries of western blotting in COL1 and αSMA. [file 12890_2021_1712_MOESM1_ESM.pdf]

Supplementary Figure 1

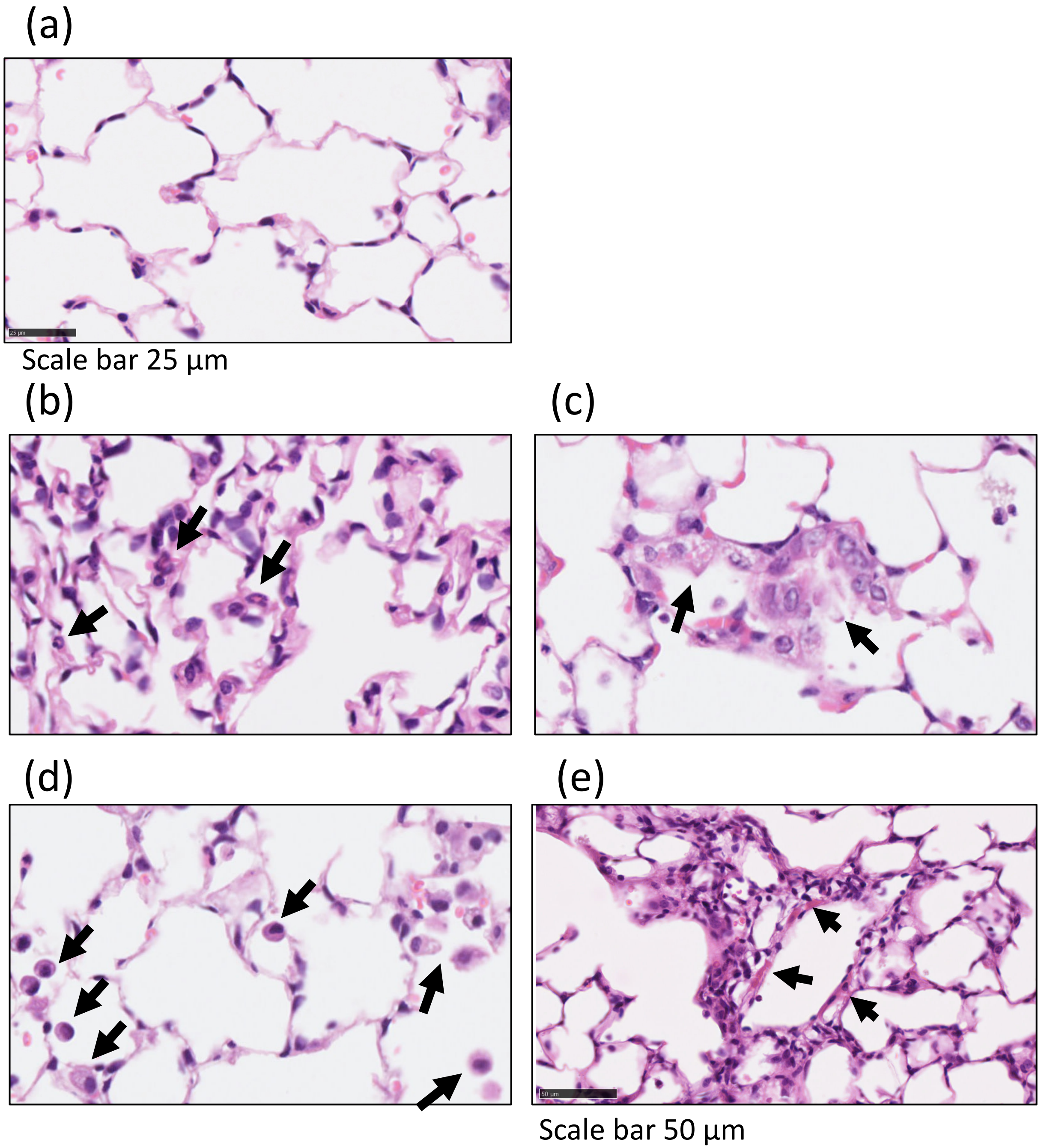


Time course of histological changes in the Lung Injury model created by intratracheal bleomycin at 1mg/kg.
(a) Normal lung tissue section, HE stain. (magnification x400).
(b) Lung tissue HE stain on day 2 after intratracheal bleomycin administration. The alveolar interstitum are thickened, and there is neutrophilic infiltration (arrows) in the alveoli and interstitium (magnification x400). The number of neutrophil is less than bacterial pneumonia.
(c) HE stained image on day 4 after bleomycin administration. Interstitial edema is reduced. Protein-rich debris with cells can be seen in the alveoli (arrow) (magnification x400).
(d) HE stained image on day 7 after bleomycin administration. The immune cells in the alveoli are mainly macrophages (arrow) (magnification x400).
(e) Day 7. Hyaline membrane formation is observed (arrows) (magnification x200).

# Supplementary Figure 1

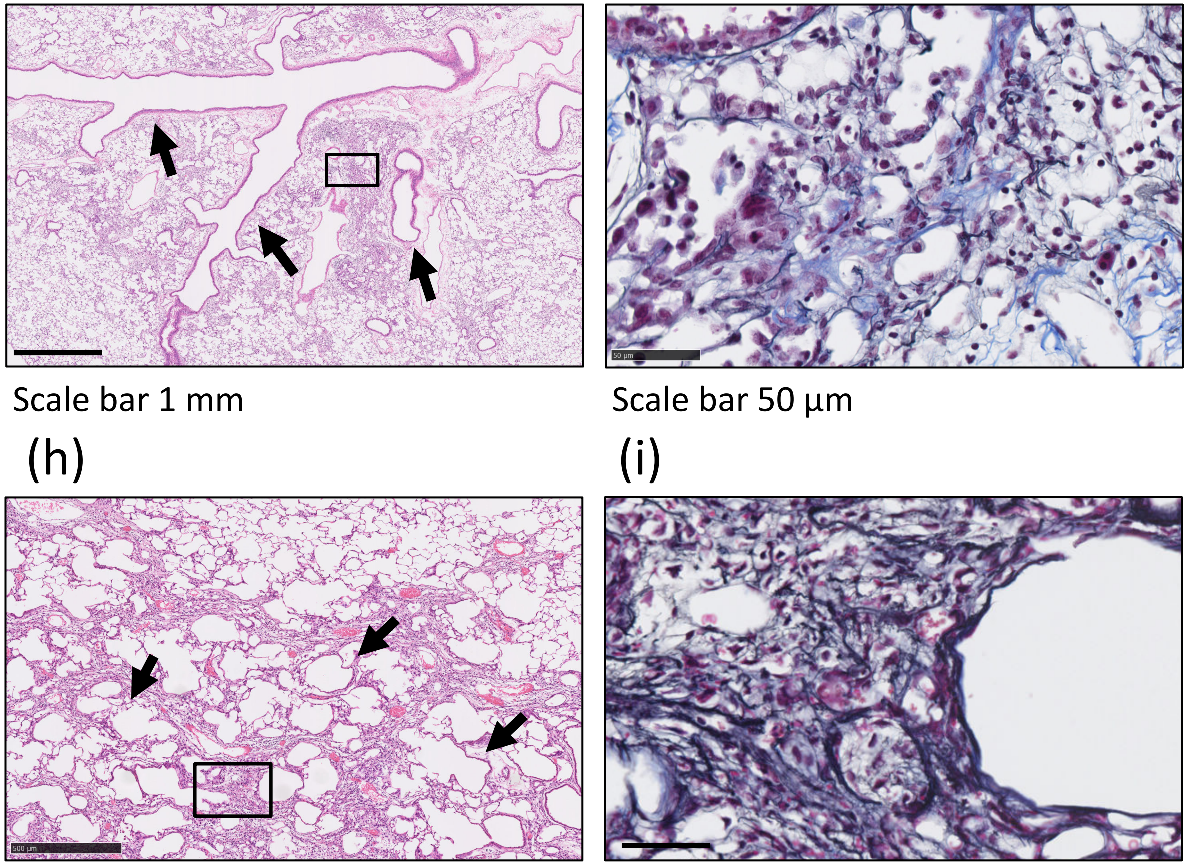

(f) Scale bar 1 mm

(g) Scale bar 50 µm

(h) Scale bar 500 µm

(i) Scale bar 50 µm

(f) On day 21, HE staining (x20). Traction bronchiectasis is seen (arrow).
(g) Inside the left rectangle, Elastica Masson stain (x200). Extracellular matrix hyperplasia (blue) is seen in the alveolar interstitium.
(h) On day 35, HE stain (x40). Destruction of alveolar structures is seen, with honeycombed changes (arrows).
(i) Inside the left rectangle, Elastica Masson stain (x200). The extracellular matrix of the alveolar interstitium has been replaced by fibrous tissue, which is seen as black bundle.

Supplementary Figure 2

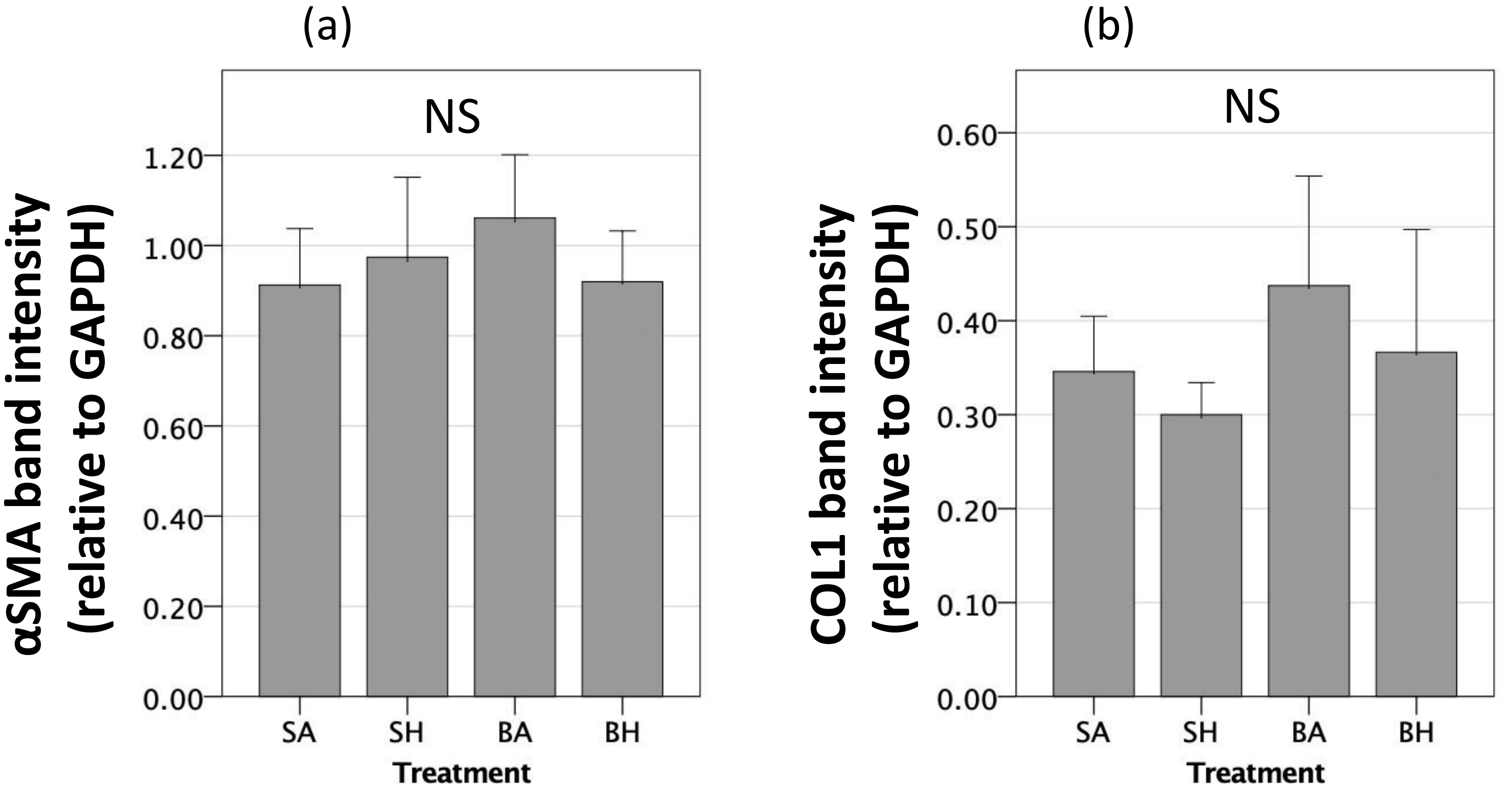


Western blotting densitometry of COL1 and αSMA, which band images are shown in Figure 4. (a) αSMA. There was no significant difference in the band intensity of αSMA(relative to GAPDH) between treatment groups (SA 0.91[95%CI:0.79-1.04], SH 0.97[95%CI:0.8-1.15], BA 1.06[95%CI:0.92-1.2], BH 0.92[95%CI:0.81-1.03]). (b) COL1. There was no significant difference in the band intensity of COL1(relative to GAPDH) between treatment groups (SA 0.35[95%CI:0.29-0.4], SH 0.3[95%CI:0.27-0.33], BA 0.44[95%CI:0.32-0.55], BH 0.37[95%CI:0.24-0.5]).
