# Supplementary material for: The effects of inhaling hydrogen gas on macrophage polarization, fibrosis, and lung function in mice with bleomycin-induced lung injury
Source: BMC Pulm Med. 2021 Oct 31;21:339. doi: 10.1186/s12890-021-01712-2 (PMC8559370; doi:10.1186/s12890-021-01712-2)
Supplement: Supplementary file 2 — Additional file 2: Table S1. Information on the number and treatments of samples in the analysis; Table S2. Primer summery; Table S3. Antibody summery. [file 12890_2021_1712_MOESM2_ESM.pdf]

Supplementary Table 1

	Group	Intratracheal administration	Inhaled Gas	Inhalation period	Mice, total	Excluded (humane endpoint)	N (Included in analysis)	
Mice 21 days after administration	SA	Saline	Air	21 days	6	0	6	Respiratory physiological examination(Fig1)<br>Computed tomography(Fig2)<br>HE and E-M staining, histological scoring(Fig3)<br>Western blotting(Fibronectin, αSMA, COL1)(Fig4)
	SH	Saline	3.2% H_2	21 days	6	0	6	
	BA	Bleomycin	Air	21 days	13	2	11	
	BH	Bleomycin	3.2% H_2	21 days	13	2	11	
Mice 7 days after administration	SA	Saline	Air	7 days	12	0	12	RT-PCR(Fig5),<br>-SA:12, SH:12, BA:28, BH:28<br><br>Western blotting, Immunostaining (TGFβ, IL6)(Fig6),<br>BALF assay(Figure 7-a,b)<br>Immunofluorescence (Iba-1, CD 163) (Fig8)<br>-SA:6, SH:6, BA:15, BH:15
	SH	Saline	3.2% H_2	7 days	12	0	12	
	BA	Bleomycin	Air	7 days	28	0	28	
	BH	Bleomycin	3.2% H_2	7 days	28	0	28	
Mice 7 days after administration (Additional experiments)	BA	Bleomycin	Air	7 days	10	0	10	BALF cells RT-PCR (Fig 6-d)
	BH	Bleomycin	3.2% H_2	7 days	10	0	10	
Total					138	4	134	

Termination method	Subjects	n
CO_2 asphyxiation	Euthanized for meeting humane endpoint, and excluded from analysis	4
Exsanguination under deep anesthesia	subject of analysis (before scheduled sampling)	134

Supplementary Table 2

**Primer summery**		
IL-6	Forward	GAGGATACCACTCCCAACAGACC
	Reverse	AAGTGCATCATCGTTGTTCATACA
IL-4	Forward	AGATGGATGTGCCAAACGTCCTCA
	Reverse	AATATGCGAAGCACCTTGGAAGCC
IL-13	Forward	TGAGGAGCTGAGCAACATCACACA
	Reverse	TGCGGTTACAGAGGCCATGCAATA
IL-10	Forward	CGGGAAGACAATAACTGCACCC
	Reverse	CGGTTAGCAGTATGTTGTCCAGC
COL1	Forward	ATCTCCTGGTGCTGATGGAC
	Reverse	ACCTTGTTTGCCAGGTTCAC
Fibronectin	Forward	CGAGGTGACAGAGACCACAA
	Reverse	CTGGAGTCAAGCCAGACACA
RPL4	Forward	GCCAAGACTATGCGCAGGAAT
	Reverse	GTAGCTGCTGCTTCCAGCTT

Supplementary Table 3

**Antibody summery**		
Target (Host, Class)	Company, Catalog no	Dilution
Fibronectin (Rabbit polyclonal)	Proteintec 15613-1-AP	1:1,000 (WB)
αSMA (Mouse monoclonal)	Abcam ab7817	1:3,000 (WB)
COL1 (Rabbit polyclonal)	Abcam ab34710	1:2,000 (WB)
TGFβ (Rabbit monoclonal)	Abcam ab215715	1:10,000 (WB)
βactin (Mouse monoclonal)	Sigma A5441	1:10,000 (WB)
GAPDH (Mouse monoclonal)	Proteintec 60004-1-Ig	1:5,000 (WB)
Mouse IgG labeling HRP (Goat)	Jackson ImmunoResarch 115-035-062	1:10,000 (WB)
Rabbit IgG l labeling HRP (Goat)	Jackson ImmunoResarch 111-035-144	1:10,000 (WB)
TGFβ (Rabbit polyclonal)	Abcam ab92486	1:200 (IHC)
IL-6 (mouse monoclonal)	Abcam ab9324	1:500 (IHC)
Mouse IgG labeling biotin (Goat)	Jackson ImmunoResarch 115-065-146	1:1,000 (IHC)
Rabbit IgG labeling biotin (Goat)	Jackson ImmunoResarch 115-065-144	1:1,000 (IHC)
Iba-1 (Goat monoclonal)	Abcam ab5076	1:200 (IF)
CD163 (Rabbit monoclonal)	Abcam ab182422	1:400 (IF)
Goat IgG AlexaFlour 488 (Donkey)	Abcam ab150133	1:400 (IF)
Rabbit IgG AlexaFlour 594 (Donkey)	Abcam ab150064	1:400 (IF)
