# Supplementary material for: The effects of inhaling hydrogen gas on macrophage polarization, fibrosis, and lung function in mice with bleomycin-induced lung injury
Source: BMC Pulm Med. 2021 Oct 31;21:339. doi: 10.1186/s12890-021-01712-2 (PMC8559370; doi:10.1186/s12890-021-01712-2)
Supplement: Supplementary file 3 — Additional file 3. The original, full-length western blot images of fibronectin, αSMA, GAPDH, COL1, TGF-β1 and β-actin. [file 12890_2021_1712_MOESM3_ESM.doc]

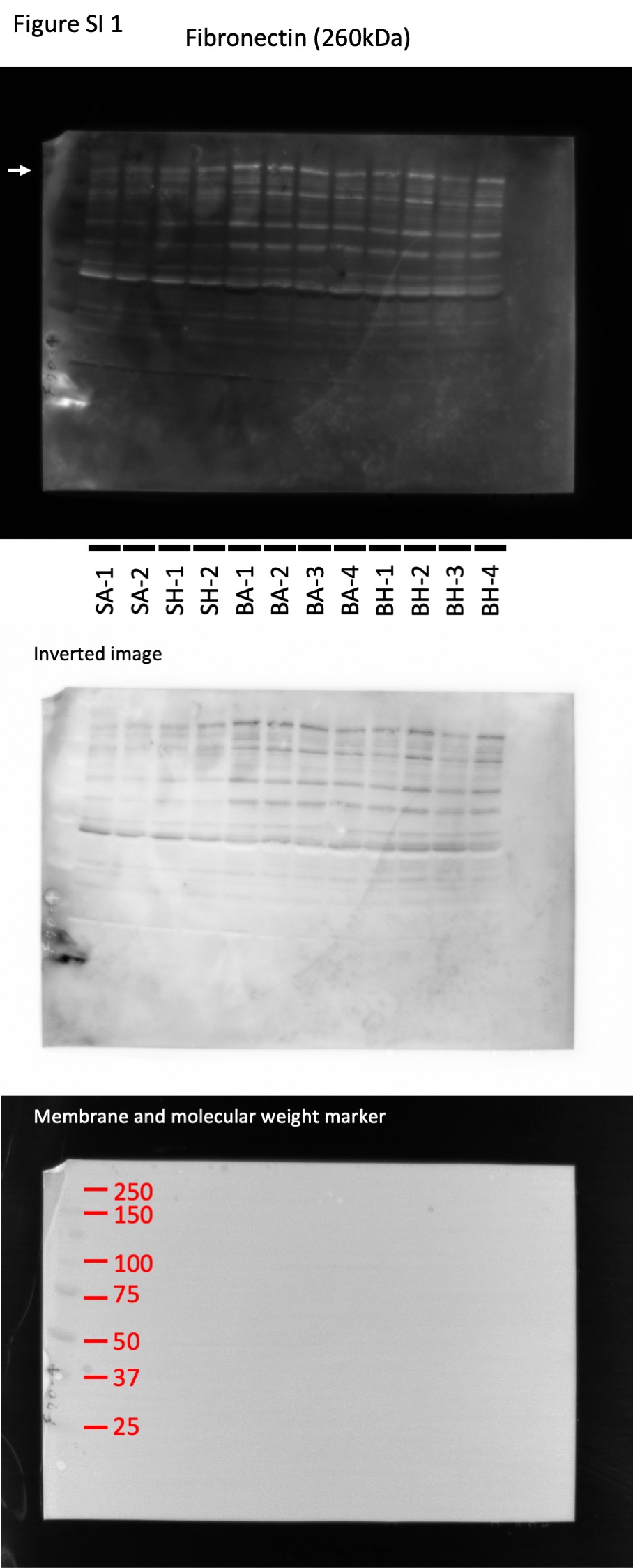


Figure SI 1: Original, full-length images of western blot for Fibronectin levels in right lobes, taken from the bleomycin induced-lung injury model with hydrogen inhalation for 21 days. The inverted and cropped image is shown in Figure 4-a. The blot bands of fibronectin (the molecular weight is 260kDa) is indicated by white arrow. Top figure shows unprocessed original image, middle figure shows the inverted image, and bottom figure shows membrane and molecular weight marker. SA, saline administration, and air inhalation; SH, saline administration, and hydrogen inhalation; BA, bleomycin administration and air inhalation; BH, bleomycin administration and hydrogen inhalation.


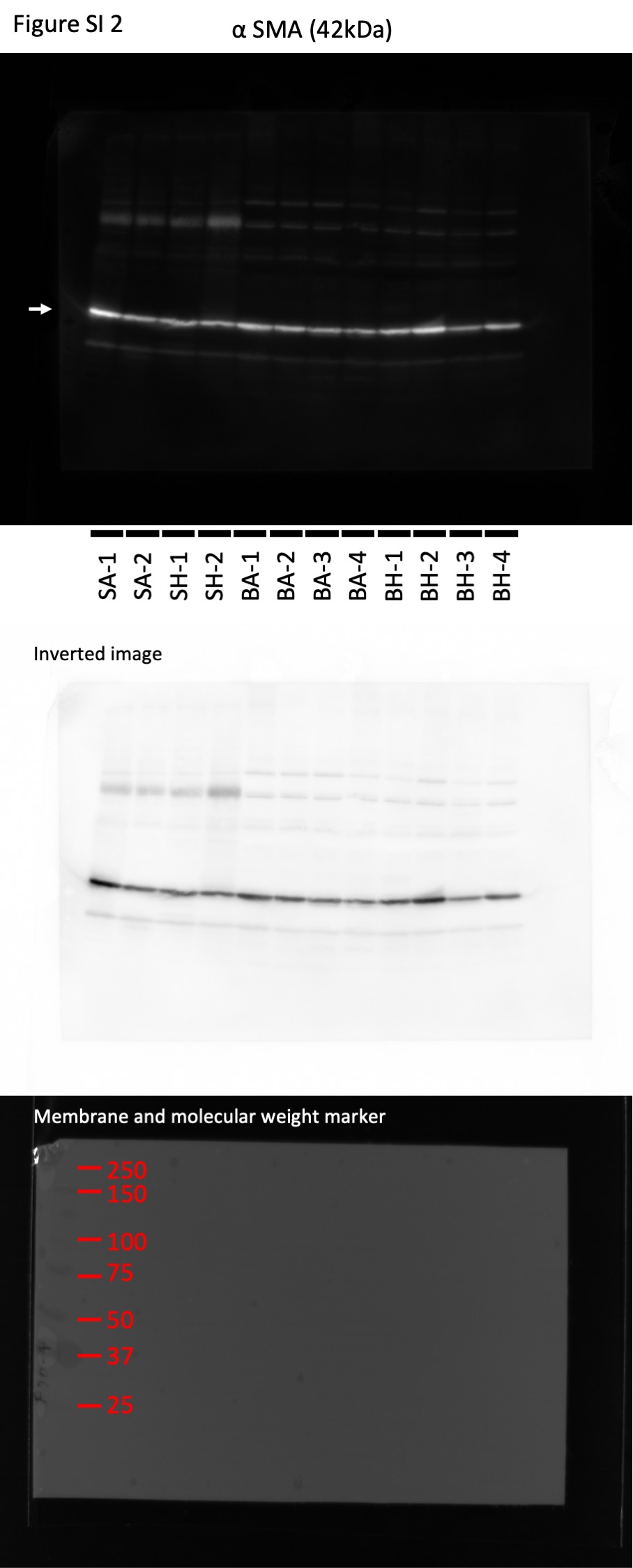


Figure SI 2: Original, full-length images of western blot for αSMA levels in right lobes, taken from the bleomycin induced-lung injury model with hydrogen inhalation for 21 days. The inverted and cropped image is shown in Figure 4-a. The blot bands of αSMA (the molecular weight is 42kDa) is indicated by white arrow. Top figure shows unprocessed original image, middle figure shows the inverted image, and bottom figure shows membrane and molecular weight marker. SA, saline administration, and air inhalation; SH, saline administration, and hydrogen inhalation; BA, bleomycin administration and air inhalation; BH, bleomycin administration and hydrogen inhalation.


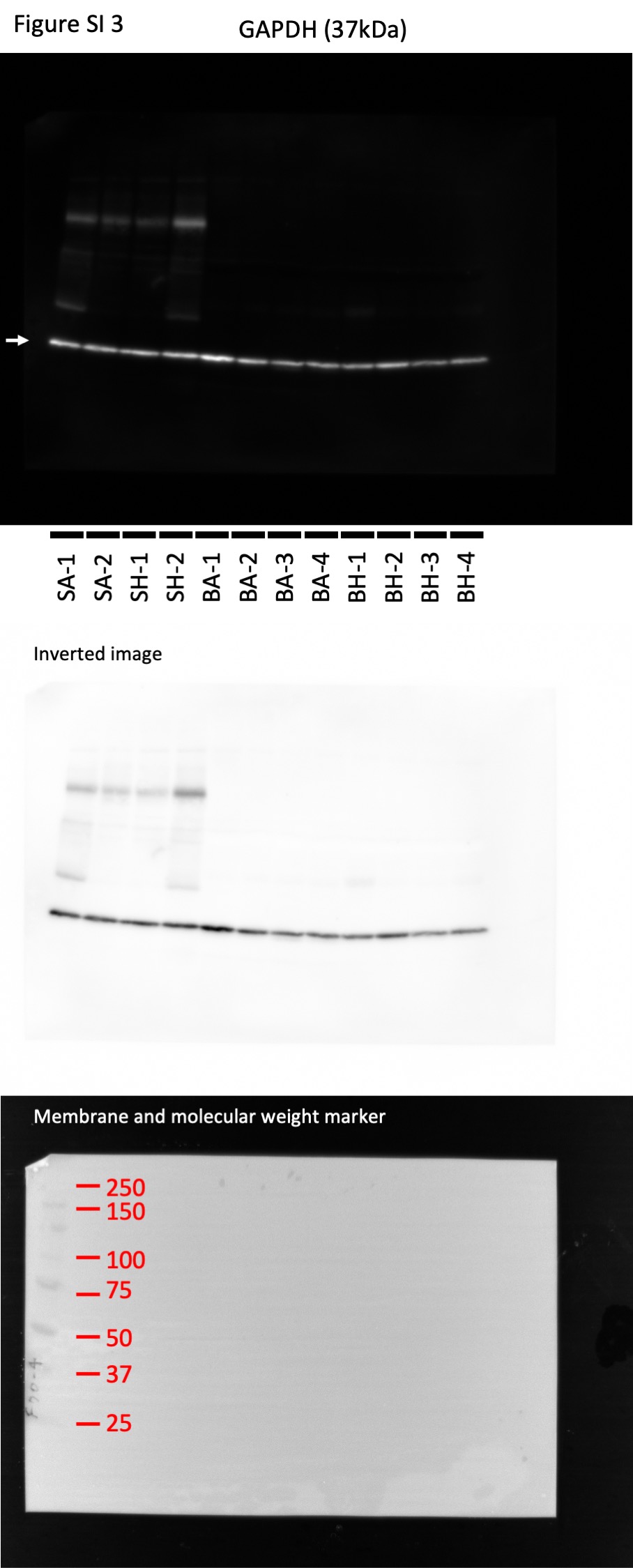


Figure SI 3: Original, full-length images of western blot for GAPDH levels in right lobes, taken from the bleomycin induced-lung injury model with hydrogen inhalation for 21 days. The inverted and cropped image is shown in Figure 4-a. The blot bands of GAPDH (the molecular weight is 37kDa) is indicated by white arrow. Top figure shows unprocessed original image, middle figure shows the inverted image, and bottom figure shows membrane and molecular weight marker. SA, saline administration, and air inhalation; SH, saline administration, and hydrogen inhalation; BA, bleomycin administration and air inhalation; BH, bleomycin administration and hydrogen inhalation.


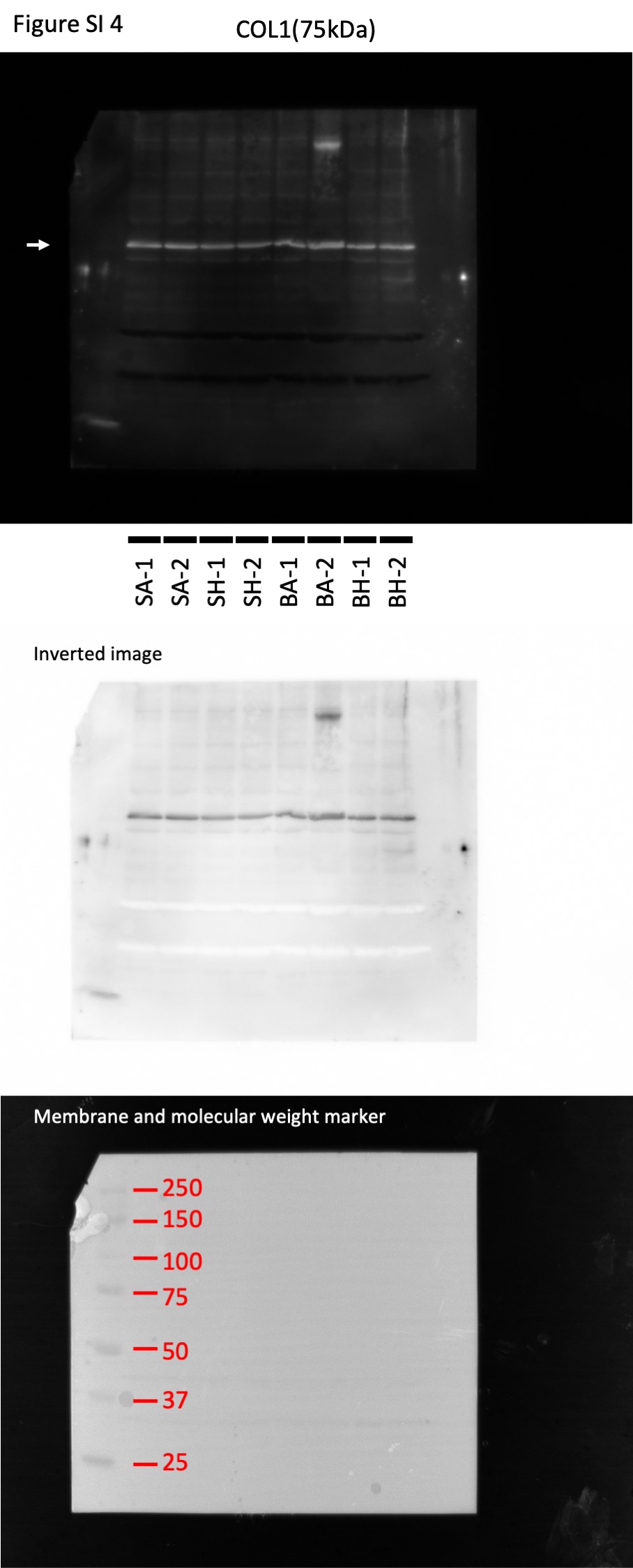


Figure SI 4: Original, full-length images of western blot for COL1 levels in right lobes, taken from the bleomycin induced-lung injury model with hydrogen inhalation for 21 days. The inverted and cropped image is shown in Figure 4-a. The blot bands of COL1 (the molecular weight is 75kDa) is indicated by white arrow. Top figure shows unprocessed original image, middle figure shows the inverted image, and bottom figure shows membrane and molecular weight marker. SA, saline administration, and air inhalation; SH, saline administration, and hydrogen inhalation; BA, bleomycin administration and air inhalation; BH, bleomycin administration and hydrogen inhalation.


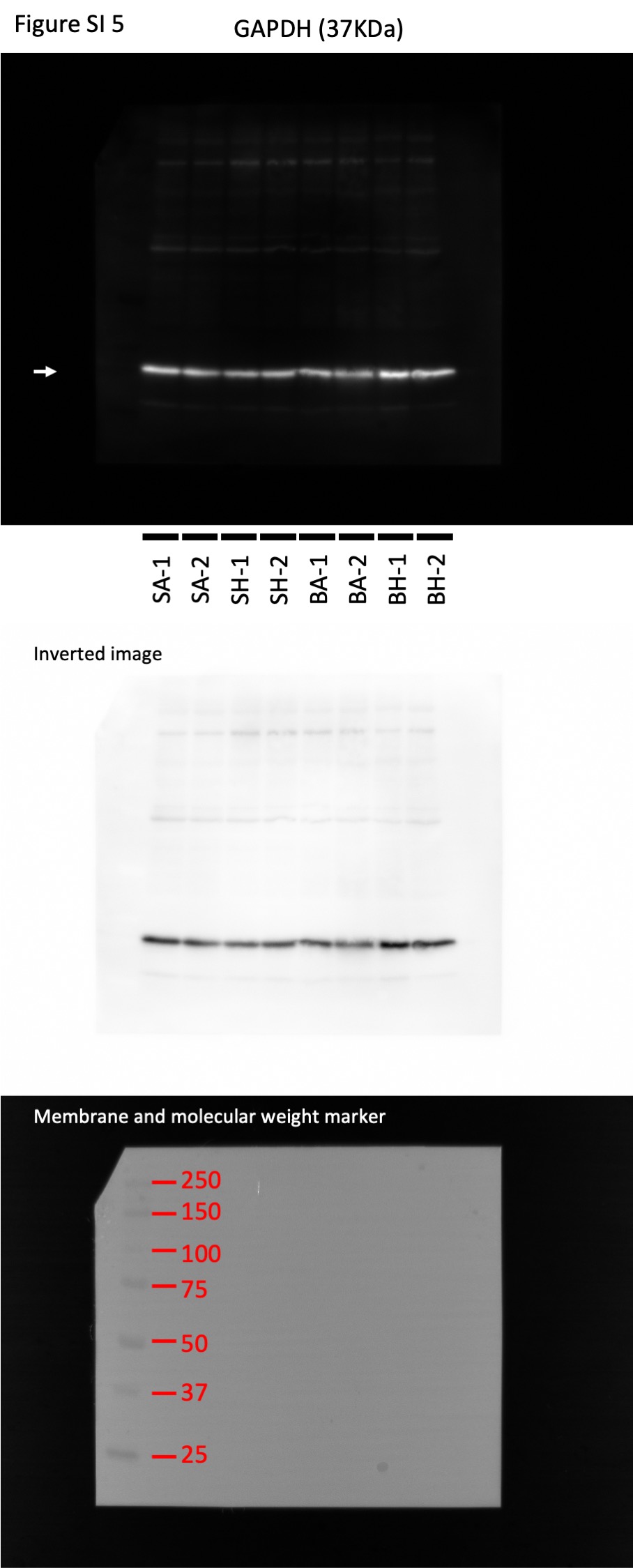


Figure SI 5: Original, full-length images of western blot for GAPDH levels in right lobes, taken from the bleomycin induced-lung injury model with hydrogen inhalation for 21 days. The inverted and cropped image is shown in Figure 4-a. The blot bands of GAPDH (the molecular weight is 37kDa) is indicated by white arrow. Top figure shows unprocessed original image, middle figure shows the inverted image, and bottom figure shows membrane and molecular weight marker. SA, saline administration, and air inhalation; SH, saline administration, and hydrogen inhalation; BA, bleomycin administration and air inhalation; BH, bleomycin administration and hydrogen inhalation.


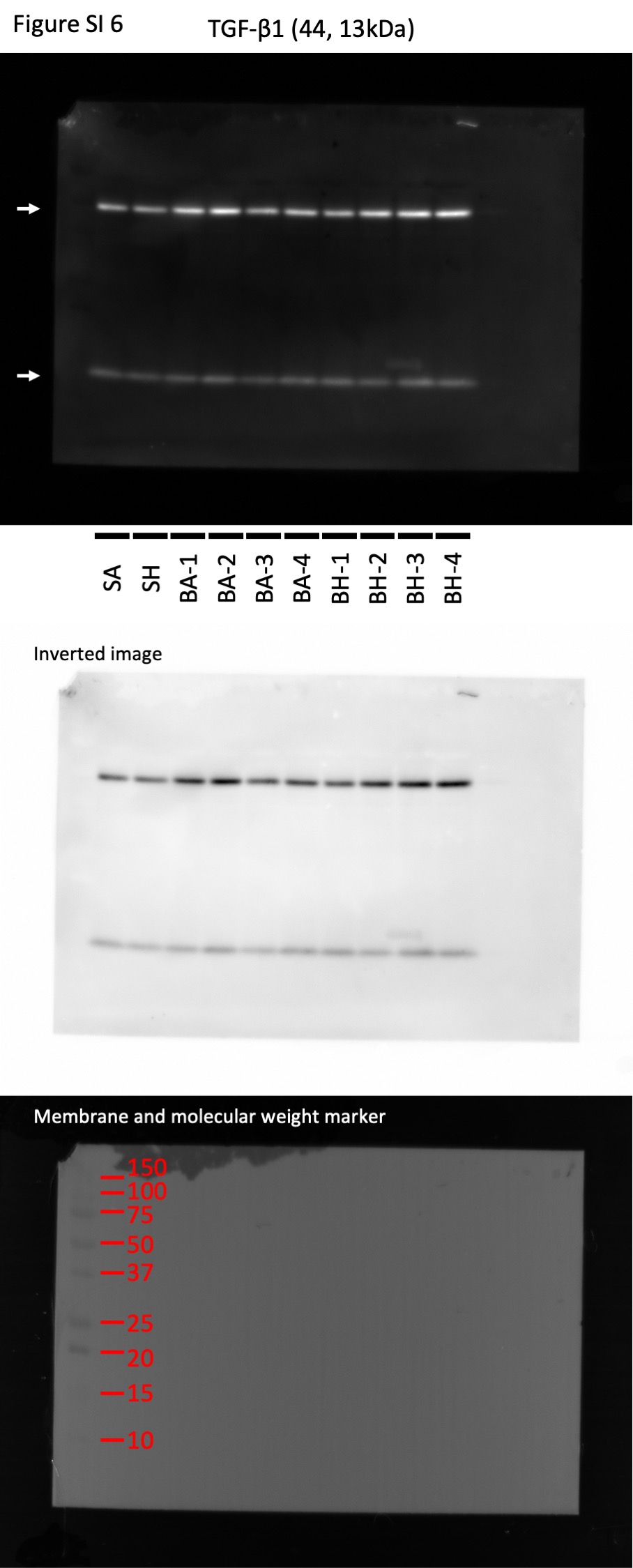


Figure SI 6: Original, full-length images of western blot for TGF-β1 levels in right lobes, taken from the bleomycin induced-lung injury model with hydrogen inhalation for 7 days. The inverted and cropped image is shown in Figure 6-a. The blot bands of TGF-β1 (the molecular weight is 44kDa and 13kDa) is indicated by white arrow. Top figure shows unprocessed original image, middle figure shows the inverted image, and bottom figure shows membrane and molecular weight marker. SA, saline administration, and air inhalation; SH, saline administration, and hydrogen inhalation; BA, bleomycin administration and air inhalation; BH, bleomycin administration and hydrogen inhalation.


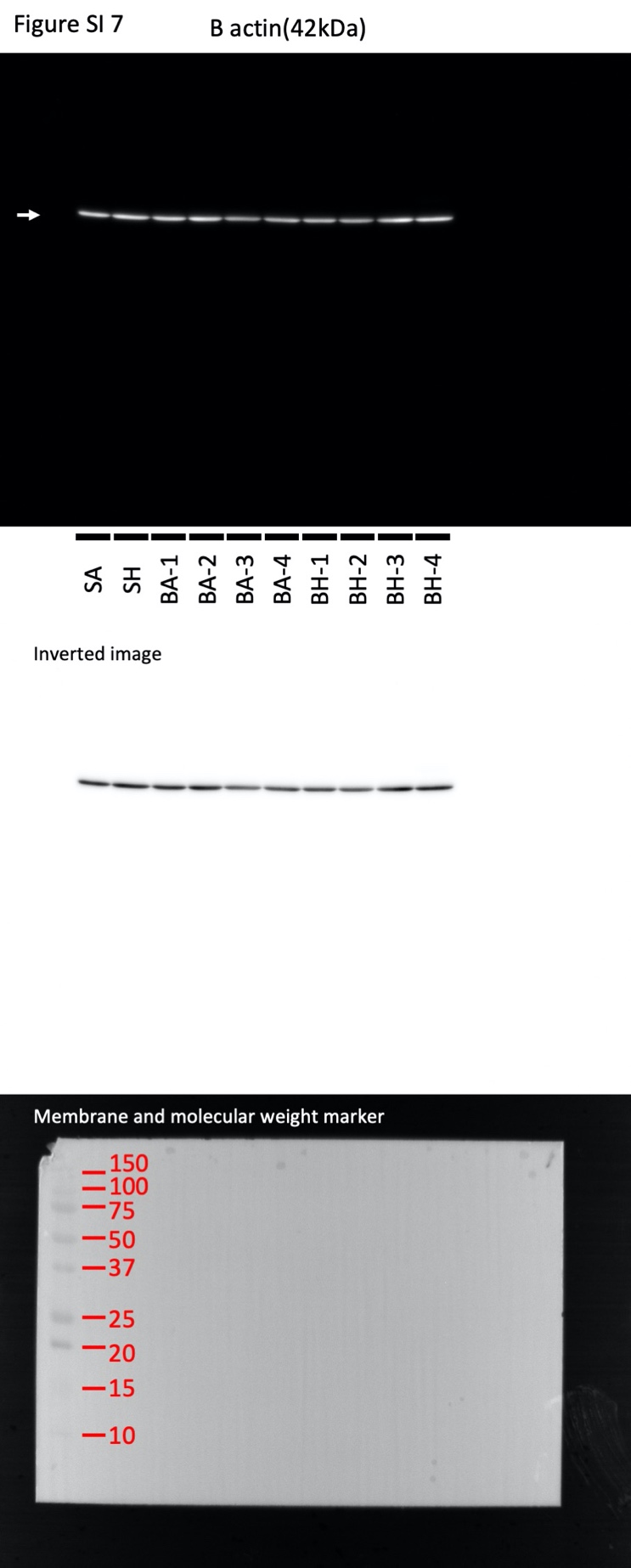


Figure SI 7: Original, full-length images of western blot for β-actin levels in right lobes, taken from the bleomycin induced-lung injury model with hydrogen inhalation for 7 days. The inverted and cropped image is shown in Figure 6-a. The blot bands of β-actin (the molecular weight is 42Da) is indicated by white arrow. Top figure shows unprocessed original image, middle figure shows the inverted image, and bottom figure shows membrane and molecular weight marker. SA, saline administration, and air inhalation; SH, saline administration, and hydrogen inhalation; BA, bleomycin administration and air inhalation; BH, bleomycin administration and hydrogen inhalation.
